# Pronoun interpretation in Mandarin Chinese follows principles of Bayesian inference

**DOI:** 10.1371/journal.pone.0237012

**Published:** 2020-08-19

**Authors:** Meilin Zhan, Roger Levy, Andrew Kehler

**Affiliations:** 1 Department of Brain and Cognitive Sciences, Massachusetts Institute of Technology, Cambridge, MA, United States of America; 2 Linguistic Department, University of California San Diego, La Jolla, CA, United States of America; University of Toronto, CANADA

## Abstract

Successful natural language understanding requires that comprehenders be able to resolve uncertainty in language. One source of potential uncertainty emerges from a speaker’s choice to use a pronoun (e.g., he, she, they), since pronouns often do not fully specify the speaker’s intended referent. Nevertheless, comprehenders are typically able to interpret pronouns rapidly despite having limited cognitive resources. Here we report three pronoun interpretation experiments that investigate whether comprehenders reverse-engineer a speaker’s referential intentions based on Bayesian principles, as documented in previous studies for English. Using Mandarin Chinese, we test the generality of the Bayesian pronoun interpretation theory, and further evaluate the predictions of the theory in ways that are not possible in English. Our results lend both qualitative and quantitative support to a cross-linguistically general Bayesian theory of pronoun interpretation.

## Introduction

Ambiguity abounds in natural language, and yet comprehenders are typically able to recover the intended messages of their interlocutors. A common source of ambiguity emerges from a speaker’s choice to use a pronoun (e.g. *he*, *she*, *they*, etc.), as oftentimes pronouns carry too little information to fully specify the intended referent. Despite the speaker having opted for an ambiguous expression in lieu of a less ambiguous or unambiguous one (e.g., a definite description or proper name), comprehenders are typically able to resolve pronouns with seemingly minimal interpretive effort. The obvious question is how they manage to do this.

According to a commonly-held view, one that we take to have been implicitly assumed in much of the literature on pronominal reference for decades, there is a singular notion of entity salience or prominence that underlies pronoun usage. In essence, on this theory, speakers use a pronoun when referring to a salient referent, and addressees will hence use the same cues to salience to successfully identify the referent. For instance, in their well-known theory relating referential form to cognitive status, Gundel et al. [1] ask *“What do speakers/writers know that enables them to choose an appropriate form to refer to a particular object and what do readers/hearers know that enables them to identify correctly the intended referent of a particular form?*”. They answer their question with a set of cognitive statuses that mediate between production and interpretation for a variety of referential forms across languages; the status of in focus corresponds to personal pronouns. Competing analyses (e.g., [2, 3]) are founded on the same assumption. (Note: A reviewer correctly points out that much of the classic research on pronouns in psycholinguistics focuses primarily on interpretation, without being explicit about its assumptions concerning the relationship between interpretation and production. Whereas it is tempting to surmise that the field took the singular salience model as being sufficiently obvious so as to not warrant comment, it is possible that the question was simply not considered. In that case, our work can be seen as advocating for a particular structure on this relationship; one that contradicts the idea that there is a singular notion of salience that mediates between production and interpretation, as we describe below.) The task for researchers who study reference then amounts to identifying the different factors that contribute to entity salience and the manner in which they combine. Previous work has proposed a variety of such factors, including but not limited to grammatical role [4], first-mention [5], grammatical parallelism [6], thematic role [7, 8], information structure [9, 10], semantics [11], and world knowledge [12].

A recently-proposed Bayesian model [13, 14], on the other hand, runs counter to that view. This model proposes that comprehenders reverse-engineer speakers’ intended referents following Bayesian principles, according to which an addressee interprets a pronoun by combining their estimate of the probability that the speaker is going to mention a particular entity next at a particular point in the discourse, independent of the form of reference they will use (represented as the prior term in Bayes’ Rule), with their estimates of the probability that they would use a pronoun to mention this entity (the production model, represented as the likelihood term in Bayes’ Rule). The model therefore implies a dissociation between production and interpretation, since the contextual factors that condition the prior will only be brought to bear for interpretation and not necessarily production. Despite the counterintuitiveness of this dissociation, several studies have confirmed the predictions of the model providing a range of English data produced by native speakers [10, 14–16] as well as second language speakers [17].

Relatively little work, however, has investigated whether the predictions of the theory generalize across different languages. The primary goal of this paper is to remedy this, by testing the generality of the Bayesian theory on pronominal reference in Mandarin Chinese. Mandarin is chosen not only for its having a different system of referential forms than English, but also due to the existence of syntactic alternations—specifically canonical active, *ba*-active, and *bei*-passive variants—that will allow us to examine predictions that are not possible to test in English. We adopted a passage completion paradigm [7, 14, 15] that allows us to measure and tease apart biases associated with pronoun interpretation, production, and prior next-mention biases. We provide both qualitative and quantitative model evaluations for the experimental data.

### Overview of the Bayesian model

As mentioned above, the Bayesian model posits that when interpreting a pronoun, comprehenders reverse-engineer the speaker’s intended referent following Bayesian principles [10, 13, 14]. Eq (M1) captures the relationship between interpretation and production on the model, following Bayes’ Rule from normative probability theory:

$$\begin{array}{c} P\left(\text{referent}|\text{pronoun}\right)=\frac{P(\text{pronoun}|\text{referent})P(\text{referent})}{\;\sum_{\text{referent}\in \text{possible}\;\text{referents}}\;P\left(\text{pronoun}|\text{referent}\right)P\left(\text{referent}\right)} \end{array}$$ (M1)

The posterior term *P*(referent|pronoun) represents the pronoun interpretation bias: upon hearing a pronoun (e.g., *he*), the probability that the comprehender will resolve it to a particular referent. On the other hand, the likelihood term *P*(pronoun|referent) represents the pronoun production bias: the probability of the speaker using a pronoun to refer to an intended referent. Finally, the prior term *P*(referent) denotes the next-mention bias: the probability that a specific referent gets mentioned next by the speaker, regardless of the form of referring expression that she chooses. On this model, therefore, pronoun interpretation biases result from an interpreter integrating his ‘top-down’ predictions about the content of the ensuing message (particularly, who gets mentioned next) with the ‘bottom-up’ linguistic evidence (particularly, the fact that the speaker opted to use a pronoun).

Kehler et al.’s Bayesian account comes in two varieties. As it stands, Eq (M1) says only that the relationship between pronoun interpretation and pronoun production follows Bayesian principles, without further specifying the types of contextual factors that affect each term in the numerator. We refer to this claim as the weak form of the Bayesian hypothesis. The weak analysis therefore simply predicts that if independent estimates of the prior, likelihood, and posterior probabilities were obtained, Eq (M1) would approximately hold. Kehler et al. also suggested, however, that the two terms in the numerator of Eq (M1) are conditioned by different types of contextual factors. On the one hand, they noted that the data they surveyed suggested that the factors that condition the next-mention bias *P*(referent) are primarily semantic and pragmatic in nature (e.g., verb type and coherence relations; see below). On the other hand, the factors that condition the production bias *P*(pronoun|referent) appear to be grammatical and/or information structural (e.g., based on grammatical role obliqueness or topichood, both of which amount to a preference for sentential subjects). The resulting prediction, therefore, is that a speaker’s decision about whether or not to pronominalize a reference will be insensitive to a set of semantic and pragmatic contextual factors that the comprehender will nonetheless bring to bear in interpretation. We refer to this as the strong form of the hypothesis.

There is evidence to support the strong hypothesis. We illustrate with the results of a passage completion study [10] (see also [16]). The contexts they employed, as well as those used in the experiments reported on herein, utilize implicit causality (IC) contexts, so we start with describing those [11, 13, 18–22]. IC verbs are so-called because they are said to impute causality to one of the participants associated with the event they denote, which in turn affects subsequent referential biases. For instance, if participants in a passage completion task are presented with a prompt like (1-a),

(1)
  a Amanda amazed Brittany because she ________
  b Amanda detested Brittany because she ________

the large majority of completions will point to Amanda as the pronominal referent. After all, Amanda must be amazing, and hence one expects to hear why. Because causality is imputed to the subject, verbs like *amaze* are called subject-biased IC verbs. If participants are given a prompt like (1-b), on the other hand, the large majority of completions will point to Brittany as the pronominal referent. After all, Brittany must be detestable, and hence one expects to hear why. Because causality is imputed to the object, verbs like *detest* are called object-biased IC verbs. The existence of IC biases has been replicated repeatedly, and is hence one of the bedrock results in the field.

The experiment conducted by [10] utilized contexts such as those in (2) and (3):

(2)
  a John infuriated Bill. _____________________
  b John scolded Bill. _____________________
  c John chatted with Bill. _____________________
(3)
  a Joohn infuriated Bill. He _____________________
  b John scolded Bill. He _____________________
  c John chatted with Bill. He _____________________

The 3-way context manipulation compared subject-biased IC contexts (2-a)/(3-a), object-biased IC contexts (2-a)/(3-b), and non-IC contexts (2-c)/(3-c), and the 2-way prompt condition compared free prompts (2) with pronoun prompts (3). Participants completed the passages and judges annotated their continuations for reference.

The data collected from participants’ completions yield estimates for the different terms in Eq (M1). The continuations in the free-prompt condition provide estimates for the terms on the right hand side of the equation: the participants’ choices of who to mention first in the continuation provides an estimate of the prior next-mention bias, and their choices of referring expression (pronoun or name) for the first-mentioned referent provides an estimate of the production bias. From these estimates, we can use Eq (M1) to compute an estimated pronoun interpretation bias. This estimate can be compared against the estimates provided by the pronoun-prompt condition, which reveal the participants’ pronoun interpretation biases directly.

As predicted by the weak Bayesian hypothesis, the estimates for the interpretation bias calculated from the free prompt condition showed a strong alignment with the actual biases measured in the pronoun prompt condition, more so than for two competing models with which it was compared. Further, as predicted by the strong Bayesian hypothesis, the context manipulation affected both the next mention biases in the free condition (2) and the pronoun interpretation biases in the pronoun prompt condition (3), with subject mentions in both prompt conditions being most frequent for subject-biased IC contexts, least frequent for object-biased IC contexts, and in between for non-IC controls. However, the difference in subject next-mention rate was not coupled with a difference in pronominalization rates for subject next-mentions in the free-prompt conditions. Instead, only grammatical role of the referent’s previous mention mattered: participants pronominalized references to the previous subject far more often than ones to the previous non-subject. That is, participants were no more likely to pronominalize a mention of the previous object in an object-biased IC context like (2-b) than in a subject-biased IC one like (2-a), and similarly no more likely to pronominalize a previous-subject mention in a subject-biased context (2-a) than in an object-biased one (2-b). Whereas this result might seem counterintuitive on the sort of salience-based account described in the introduction, it is precisely what one expects on the strong form of the Bayesian analysis.

#### Coherence relations

The aforementioned study demonstrated that a semantic property, implicit causality, influences the next-mention biases for the ensuing sentence, as captured by the prior. A second factor that has been shown to influence the next-mention bias pertains to the interpreter’s expectations about what coherence relation will hold between the current and ensuing utterances. It is well known that comprehenders do not interpret adjacent clauses in a discourse independently, but instead make inferences as necessary to establish a way in which the clauses can be understood as being relevant to one another. The following are examples of specific relations that capture ways in which this relevance can be established [14, p.7]:

**Explanation**: Infer that the second sentence describes a cause or reason for the eventuality described in the first sentence.

(4) John impressed Bill. He was a fabulous young man.

**Result**: Infer that the first sentence describes a cause or reason for the eventuality described in the second sentence.

(5) John impressed Bill. He decided to hire this fabulous young man.

**Occasion**: Infer a change of state from the second sentence, taking its initial state to be the final state of the eventuality described in the first sentence.

(6) Jane went to the bookstore. She bought a pile of books.

**Elaboration**: Infer that both sentences provide descriptions of the same eventuality.

(7) Jane went to the bookstore. She took the bus to Warwick’s books on Main Street.

Hobbs [12, 23] argued that coherence establishment influences the interpretation of pronouns; for instance, the pronoun *he* in (5) will typically be taken to refer to Bill, since only on this assignment can the Result relation be established straightforwardly in light of one’s world knowledge about the relationship between impressing and hiring. Kehler and Rohde [14] subsequently argued, however, that the influence of coherence relations on pronoun interpretation is only indirect, being mediated by their effect on the prior as predicted by the strong form of the Bayesian model. A passage completion experiment reported on in [13], for instance, demonstrated that the coherence relations that participants employed in contexts containing IC verbs had a significant impact on next mention biases. For instance, in the case of subject-biased IC contexts, the rate of next mention to the previous subject was 84% for continuations that instantiated Explanation relations, but only 61% for Elaboration relations, and 10% for Result relations. The experiments described herein will analyze the effects of both verb type and coherence relations on the different components of the Bayesian analysis as applied to Mandarin Chinese.

#### Topichood

Our studies will also address an additional factor that is hypothesized to affect production biases, as represented by the likelihood term in Bayes’ Rule. Previous research on English [7, 14] has demonstrated that referents mentioned from subject position are more likely to be subsequently mentioned with pronominals as compared to those mentioned from other grammatical positions. However, these works did not isolate the source of the bias: is the bias linked specifically to grammatical role, or does it reflect a tendency to pronominalize topics (as posited by Centering Theory [9]), which are canonically mentioned in the subject position in English? Rohde and Kehler [10] investigated this question in a passage completion experiment that manipulated the voice of the context sentence:

(8)
  a Joane amazed Linda. ________________
  b Linda was amazed by Jane. ________________

The mentions of Jane and Linda occupy the same grammatical role (the subject) of their respective clauses. However, Rohde & Kehler posited that they are not equivalent in their likelihood of being the topic: whereas the subject is merely the default location to place a topic in active voice clauses (8-a), one of the motivations for using the passive is precisely to make a different event participant the topic. Because Linda is thus more likely to be the topic in (8-b) than Jane is in (8-a), the hypothesis that speakers use pronouns to remention the current topic predicts that subsequent mentions of Linda should be pronominalized at a greater rate than Jane, despite the fact that they occupy the same grammatical role. This is precisely what Rohde & Kehler found.

#### Passivization and next mention

Finally, in addition to finding the predicted effect of passivization on production biases, Rohde & Kehler also found an unanticipated effect of passivization on the prior, specifically, an increase in the next-mention bias of the demoted subject in the passive clause (e.g., Jane in (8-b)). This result is a surprise for the strong Bayesian account, since the next-mention bias is predicted to be determined by semantic factors, and hence should be insensitive to a change in syntactic structure. It is likewise a surprise for salience-based accounts of the sort mentioned in the introduction, which would predict that the effect of passivization should be *fewer* subsequent mentions for the syntactically demoted subject. Rohde & Kehler, following the explanation of a similar finding [8] for transfer-of-possession contexts, posited that the increase in mentions for the demoted subject might have an external cause—because the by-adjunct in passive clauses is optional, participants may have felt the need to remention the demoted subject to justify its inclusion in the story. We will ask if we see similar behavior in Mandarin Chinese.

### *Bei*, *Ba* and pronouns in Mandarin Chinese

We present three experiments that examine the Bayesian pronoun interpretation theory for Mandarin Chinese, in some cases exploiting particular features of the language that are not shared with English. We test whether manipulations of syntactic construction and of verb type affect the distribution of coherence relations found in the discourse continuations that participants provide, and whether and how the manipulations affect the next-mention biases, pronoun production biases, and interpretation biases revealed by the data. In this section we provide linguistic details about the syntactic constructions in Mandarin Chinese that we utilize.

#### Bei construction and affectedness

Passive voice in Mandarin Chinese is generally realized via the *bei* construction with the following linear arrangement:

(9) NP_1_ bei NP_2_ verb

In this construction, the logical object NP_1_ is in the sentence-initial position, followed by the passive marker *bei*, which introduces the logical subject NP_2_. An example of a canonical active sentence and its corresponding passive counterpart are given in (10) and (11):

(10) 美惠 打动了 洁怡.
Meihui dadong-le Jieyi.
Meihui touch-ASP Jieyi (‘Meihui touched Jieyi.’)
(11) 洁怡 被 美惠 打动了.
Jieyi bei Meihui dadong-le.
Jieyi bei Meihui touch-ASP (‘Jieyi was touched by Meihui.’)

As in English, NP_2_ is optional, per (12).

(12) 洁怡 被 打动了.
Jieyi bei dadong-le.
Jieyi bei touch-ASP (‘Jieyi was touched.’)

The meaning of *bei* in the context of a passive sentence is roughly that of ‘undergo’ or ‘experience’ [24]. The *bei* construction conveys the notion of affectedness [25], which describes an event in which an entity or person is affected physically or psychologically in some way [25, 26]. Because of this property, *bei* is often used with a resultative compound [26] composed of a morpheme signaling the action and a morpheme signaling the result of the action. In (13), for example, *jinu* is a resultative compound whereby *ji* signals the action *stimulate* and *nu* signaling the result of the action *being angry*.

(13) 张三 被 李四 激怒了.
Zhangsan bei Lisi jinu-le.
Zhangsan bei Lisi stimulate-angry-ASP (‘Zhangsan was angered by Lisi.’)

The combination of these meanings conveys the meaning of English *anger*.

The constraint requiring affectedness hence precludes cases in which the denotation of NP_1_ cannot be construed as having been been affected, as in (14):

(14) #洁怡 被 美惠 认识了.
#Jieyi bei Meihui renshi-le.
Jieyi bei Meihui know-ASP (‘Jieyi was known by Meihui.’)

Although the English translation of (14) is perfectly felicitous, the Mandarin version is not, since Jieyi is not affected by being known to Meihui. Further, in other cases the use of the passive is felicitous, but more strongly enforces a construal in which NP_1_ is viewed as affected. Consider example (15), taken from the stimuli used in our second experiment:

(15) 子豪 被 毅刚 低估了.
Zihao bei Yigang digu-le.
Zihao bei Yigang underestimate-ASP (‘Zihao was underestimated by Yigang.’)

A comprehender would more readily infer that Zihao was negatively affected upon hearing (15) than its active voice variant (*Yigang digu-le Zihao*, or *Yigang underestimated Zihao*); perhaps Yigang is Zihao’s manager and decided not to promote Zihao, for instance. As a result, because the passive *bei* construction conveys affectedness for the logical object, one might expect to see an increase in continuations describing the Result of that affectedness, which would tend to be about the logical object.

Hence *bei* passives may provide a good test case for reevaluating the finding of Rohde and Kehler [10] for English, whereby passivization yielded an increase in the next-mention bias of NP_2_ that was not predicted by the strong version of Bayesian hypothesis. A change from active voice to passive voice not only involves a change in syntactic structure, but may also affect the semantic construal of a sentence. If the *bei* construction imposes greater affectedness than the corresponding active counterpart would convey, this could in turn affect the next-mention bias.

#### Ba construction and syntactic prominence

As discussed in the previous section, because subjecthood and topichood are highly correlated in English, it is difficult to tease apart the respective influences of grammatical role and topichood without changing other properties of the context, such as the voice used. What one would like is a pair of alternative syntactic structures that are as similar as possible, but which might plausibly differ with respect to the topichood of the occupant of a particular grammatical role. The contrast in Mandarin between the canonical active voice construction and the *ba* construction provides this. It has been argued that the *ba* construction provides a way to mark a non-subject NP as a secondary topic [27] (though see [28] for arguments against this view). Since *ba* does not change the grammatical function of the argument it marks, there is a possible opportunity to reveal a potential influence of topichood on pronoun production biases, dissociated from grammatical function, as we describe in the logic below.

To facilitate our descriptions, we henceforth use NP_1_ to refer to a clause’s surface subject (which is also the logical subject in actives, but the logical object in passives), and likewise NP_2_ to refer to the clause’s surface non-subject (similarly, also the logical object in actives, but the logical subject in passives). The difference between the canonical active and *ba* constructions are shown schematically in (16):

(16)
  a NP_1_ verb NP_2_
  b NP_1_ ba NP_2_ verb

That is, unlike the active form (10), the *ba* construction places the direct object immediately after *ba* and before the verb [25]. (There are controversies about the syntactic status of the post-*ba* NP being a direct object [27, 29], but these need not concern us here.)

(17) 美惠 把 他赶走了.
Meihui ba ta gan-zou-le.
Meihui ba him kick-out-ASP (‘Meihui kicked him out.’)

One of the much discussed properties of the *ba* construction is its effect on the post-*ba* NP. For instance, as mentioned earlier, some previous research has argued that the post-*ba* NP serves as a secondary topic ([27], from which examples (18)-(20) are adapted):

(18) 张三 在 书架上 摆满了 书.
Zhangsan at book-shelf-LOC put-full-ASP book
(‘Zhangsan filled the bookshelf with books.’)
(19) 张三 把 书架 摆满了 书.
Zhangsan ba shujia bai-man-le shu.
Zhangsan ba bookshelf put-full-ASP book
(‘Zhangsan filled the bookshelf with books.’)
(20) 张三 把 书 摆满了 书架.
Zhangsan ba shu bai-man-le shujia.
Zhangsan ba book put-full-ASP bookshelf
(‘Zhangsan loaded the books onto the bookshelf.’)

On a commonly-held view in the pragmatic literature, topical elements in the sentence are those that are part of the (often implicit) question-under-discussion (QUD) to which the sentence provides an answer [30]. Tsao [27] reports that even though both (19) and (20) share the same canonical counterpart in (18), they provide answers to different questions. On the one hand, (19) is typically understood to provide an answer to question (21):

(21) What did Zhangsan do to the bookshelf?

That is, making the NP corresponding to *the bookshelf* the object of *ba* has the effect assimilating it to be part of the QUD that the utterance answers, hence making it topical. On the other hand (20) most naturally provides an answer to question (22):

(22) What did Zhangsan do to the books?

In this case, only the NP corresponding to *the books* is the object of *ba* and hence part of the QUD. The fact that the NP corresponding to *the bookshelf* occurs post-verbally means that it is part of the answer to the question and not part of the question itself, and hence is not topical. Therefore, one question we test in our experiments is whether mentions of direct objects in *ba* constructions are more likely to be pronominalized by a speaker than such mentions in a canonical active voice construction.

We note that some readers have asked whether the effect of topicalization might not alternatively be testable using the fronted-object construction common in Mandarin, which often has a topicalization interpretation, exemplified below:

(23) 这种 菜, 我 很 喜欢. 我 经常 吃.
Zhe-zhong cai, wo hen xihuan. Wo jingchang chi.
this-CL.kind dish, I very like. I frequently eat. (‘I really like this dish. I often eat it.’)

In this study, we avoided fronted-object constructions because they are likely to receive a contrastive interpretation, exemplified below:

(24) 这种 菜, 我 很 喜欢. 那种 菜, 我 不 喜欢.
zhe-zhong cai, wo hen xihuan. na-zhong cai, wo bu xihuan.
this-CL.kind dish, I very like. that-CL.kind dish, I negation like.
(‘This dish I really like. That dish I don’t like.’)

Whatever notion of topicalization might be appropriate for this contrastive interpretation, its effects on the QUD and on discourse expectations are likely to be very different than the type of topicalization under consideration. We thus eschew the use of fronted objects to minimize the possibility of contrastive interpretations influencing our data.

Finally, whereas it has been claimed that *ba*, like *bei*, imposes an affectedness requirement on its logical object [24, 25], the nature of this constraint is not entirely clear. For example, whereas the *bei*-passive in (25) is not felicitous, the *ba*-active variant in (26) is fine.

(25) #房间 被 我 打扫了 一 下.
#Fangjian bei wo dasao-le yi-xia.
Room bei I sweep-ASP one-CL (‘The room was swept by me.’)
(26) 我 把 房间 打扫了 一 下.
Wo ba fangjian dasao-le yi-xia.
I ba room sweep-ASP one-CL (‘I swept the room.’)

These examples and others suggest that insofar as the *ba* construction might require a construal of affectedness for the post-*ba* NP, this requirement is milder than for the *bei* construction. We will thus focus on *bei* passives when examining model predictions regarding affectedness, and utilize *ba* constructions for examining the effect of being a secondary topic.

### Previous work on pronoun interpretation and referent predictability in Mandarin

Unlike English, Mandarin Chinese is a null-pronoun language, in which pronouns come in both null (unpronounced) and overt forms. For example, one can felicitously respond to (27) using either a null pronoun (∅) as in (28-a) or an overt pronoun (*nimen*/“you”) as in (28-b).

(27) 昨天 我 和 张三 去 钓鱼.
Zuotian wo he Zhangsan qu diaoyu.
Yesterday I with Zhangsan go fish (‘Yesterday Zhangsan and I went fishing.’)
(28)
  a ∅ 钓 着 ∅ 了 吗?
  ∅ Diao zhao ∅ le ma?
  (You) Catch succeed (fish) ASP Q
  ‘Did (you) catch (any fish)?’
  b 你们 钓 着 鱼 了 吗?
  Nimen diao zhao yu le ma?
  You catch succeed fish ASP Q (‘Did you catch any fish?’) (Examples adapted from [25])

As shown in (28-a), null pronouns may potentially occur in the subject position and/or the object position. They are also compatible with all values for person and number; Mandarin provides no disambiguating agreement information on verbs.

Several studies have analyzed the behavior of Mandarin referential expressions that utilize the different syntactic contexts surveyed. Yang et al. [31], for instance, report on four experiments that examine the varying effects of using null pronouns, overt pronouns, and repeated names on reading times in brief Mandarin discourses. In contrast to the studies reported on here, their study did not examine interpretation biases directly, but instead asked whether a speaker’s choice to use a particular type of referring expression would affect reading times, similar to the ‘repeated name penalty’ effect found in previous work on English [32]. Consistent with the English results, penalties were found for repeated names when compared to both null and overt pronouns, specifically when the referring expression and antecedent were both mentioned from the subject position of their respective sentences. Of particular interest is the fact that null and overt pronouns displayed no significant differences with respect to a repeated name effect; subject-to-subject references with overt pronouns were read just as swiftly as those with null pronouns. This suggests a degree of interoperability between null and overt pronouns, with each being associated with similar referential biases. (Further support for such interoperability was provided by a corpus study reported on by [1]. Gundel et al. annotated a collection of referring expressions for the six categories on their Givenness Hierarchy of cognitive status, and found that both null and overt pronouns were predominantly found with referents that held the highest possible status (i.e., in focus). Experimental work on Korean [33] and Japanese [34] also found that null and overt pronouns are associated with similar biases in their respective languages. This stands in contrast to studies of certain other languages, such as Italian [35], that suggest that a ‘division of labor’ exists between the two forms, whereby overt pronouns tend to favor referents other than what would have been the preferred referent had a null pronoun been used instead.)

Yang et al. [36] followed up by conducting a set of self-paced reading time experiments designed in part to examine the role of syntactic construction during pronoun interpretation. Their Experiment 3 employed canonical active, *ba*-active, and *bei*-passive contexts, and found no difference in the processing of overt pronouns between the canonical active and *ba*-active conditions which, interestingly, suggests that the preverbal object in the *ba*-active is not accorded a greater degree of salience than the post-verbal object in the canonical active. Experiment 4 then compared the processing of null pronouns in *ba*-actives and *bei*-passives, which revealed results in the passive condition consistent with those for overt pronouns in Experiment 3. (A difference was found for *ba*-actives between the two experiments, which Yang et al. attribute to the fact that their critical sentences with null subjects could be interpreted instead as VPs that are syntactically attached to the preceding context sentence. This isn’t possible in the *bei*-passive condition nor when the pronoun is overt.) As an ensemble, therefore, Yang et al.’s results suggest that the processing of null and overt pronouns in Mandarin is highly similar.

Cheng and Almor [37] report on two passage completion experiments in Mandarin that examined whether syntactic factors influence the likelihood of next mention. Both experiments utilized contexts with IC-1 verbs (particularly, Stimulus-Experiencer verbs, for which the subject and object correspond to the Stimulus and Experiencer thematic roles respectively) used in the now-familiar three syntactic constructions: canonical active, *ba*-active, and *bei*-passive (note that the original examples in [37] did not have Chinese characters; the personal names in the sentences here were created based on the phonetic information (Pinyin) from the original examples):

(29) 邓翔 激怒了付鹏, 因为/因此……
Dengxiang jinu-le Fupeng, yinwei/yinci…
Dengxiang anger-ASP Fupeng because/because-of-that…
(‘Dengxiang angered Fupeng because/, because of that…’)
(30) 邓翔 把付鹏 激怒了, 因为/因此……
Dengxiang ba Fupeng jinu-le, yinwei/yinci…
Dengxiang ba Fupeng anger-ASP because/because-of-that…
(‘Dengxiang angered Fupeng because/, because of that…’)
(31) 付鹏 被 邓翔 激怒了, 因为/因此……
Fupeng bei Dengxiang jinu-le, yinwei/yinci…
Fupeng bei Dengxiang anger-ASP because/because-of-that…
(‘Fupeng was angered by Fupeng because/, because of that…’)

Free prompts consisted of the context clause followed by the connective *yinwei* (‘because’; Experiments 1 and 2) or *yinci* (‘because of that’; Experiment 2) as shown in (29)–(31). These connectives signal the coherence relations Explanation and Result and led, as expected, to strong next-mention biases toward the subject and object referents respectively. More surprisingly, however there was a greater percentage of next-mentions of the subject than the object in *ba* contexts (30) than in their canonical active counterparts (29) across both connective conditions. That is, whereas one might have expected the syntactic promotion of the object to result in a greater number of references to the object, the opposite pattern was actually found. Note that Cheng and Almor only examined next-mention biases in these experiments, and hence did not investigate pronoun production or interpretation.

Finally, Simpson et al. [38] report on three passage completion studies intended to examine the referential biases associated with overt pronouns in Chinese, which confirmed several predictions of previous studies [39, 40] regarding the effects of grammatical role, verb semantics, and coherence relations on pronoun interpretation. However, because no free prompt condition was run alongside the overt pronoun condition, there is no way to distinguish whether these factors affected production or next-mention biases, nor how the production and next-mention biases ultimately contributed to the interpretation biases that were found. Further, all claims are based only on descriptive statistics, as no inferential statistics were provided.

### The present study

Taken together, previous work on pronoun interpretation and referent predictability in Mandarin [17, 31, 36, 38] is consistent with the idea that referential biases in Mandarin are determined in part by a variety of factors that have been previously shown to influence pronoun interpretation in English, including the syntactic form of the context sentence, the syntactic position of the antecedent, the verb semantic properties of the context sentence, and coherence relations. They do not suffice for evaluating the core tenets of the Bayesian model, however, as they do not allow for the relative contributions of the different factors to production and next-mention biases to be evaluated, nor tell us whether measured interpretation biases accord with the hypothesis that comprehenders interpret Mandarin pronouns based on Bayesian principles. The current work addresses this need, using experiments designed to systematically investigate production biases, next-mention biases, and interpretation biases via a range of syntactic and semantic manipulations, and to provide both qualitative and quantitative evaluations of the Bayesian model and competing accounts.

#### Passage completion task

The three experiments we report on all employed a passage completion task. Participants were given a context sentence and possibly a prompt for the second sentence, and asked to complete the second sentence. An example stimulus set for Experiment 1 is shown in (32)–(35).

(32) [${}_{{\text{NP}}_{1}}$ 美惠] 打动了_IC-1_ [${}_{{\text{NP}}_{2}}$ 洁怡]. __________ [IC-1, Free]
[${}_{{\text{NP}}_{1}}$ Meihui] dadong-le_IC-1_ [${}_{{\text{NP}}_{2}}$ Jieyi]. __________
Meihui touched Jieyi. __________
(33) [${}_{{\text{NP}}_{1}}$ 美惠] 打动了_IC-1_ [${}_{{\text{NP}}_{2}}$ 洁怡]. 她 __________ [IC-1, Pronoun]
[${}_{{\text{NP}}_{1}}$ Meihui] dadong-le_IC-1_ [${}_{{\text{NP}}_{2}}$ Jieyi]. Ta __________
Meihui touched Jieyi. She __________
(34) [${}_{{\text{NP}}_{1}}$ 美惠] 打动了_IC-1_ [${}_{{\text{NP}}_{2}}$ 洁怡]. __________ [IC-2, Free]
[${}_{{\text{NP}}_{1}}$ Meihui] jiegu-le_IC-2_ [${}_{{\text{NP}}_{2}}$ Jieyi]. __________
Meihui fired Jieyi. __________
(35) [${}_{{\text{NP}}_{1}}$ 美惠] 打动了_IC-1_ [${}_{{\text{NP}}_{2}}$ 洁怡]. 她 __________ [IC-2, Pronoun]
[${}_{{\text{NP}}_{1}}$ Meihui] jiegu-le_IC-2_ [${}_{{\text{NP}}_{2}}$ Jieyi]. Ta __________
Meihui fired Jieyi. She __________

Each of the three experiments features a Verb Type manipulation that compares context sentences containing IC-1 verbs (e.g., *dadong*, ‘touch’; (32) and (33)) with ones featuring IC-2 verbs (e.g., *jiegu*, ‘fire’; (34) and (35)). Context sentences always contained two event participants with stereotypical female or male names of the same gender. IC verbs were chosen so as to manipulate the prior, with IC-1 and IC-2 favoring the syntactic subject (NP_1_) and the non-subject (NP_2_) next-mentions respectively. A norming study (N = 45) was conducted prior to the three experiments to ensure that the selected verbs have a clear bias toward re-mentioning either the subject or the non-subject. A total of 50 candidate verbs were chosen based on previous studies in English [15], Spanish [41], and Chinese [42], and based on the native speaker intuitions of the first author. A passage completion task was employed that utilized stimuli like those shown in (32) and (34), in which participants wrote a complete follow-on sentence. Judges then catalogued the first-mentioned referents. From the original set, sixteen subject-biased IC-1 verbs and twenty object-biased IC-2 verbs were selected for the main experiments (see S1 Appendix for more information). For IC-2 verbs, we avoided some typical psychological verbs such as *like*, *hate* or *envy* that cannot be used with the *ba* or *bei* constructions in Mandarin.

The three experiments also include a Prompt Type manipulation that contains two levels. Stimuli in the Pronoun Prompt condition (e.g., (33) and (35)) include a context sentence and an overt pronoun that begins the second sentence. The distribution of references in the participants’ completions allows us to measure the empirical pronoun interpretation bias *P*(referent|pronoun). Stimuli in the Free Prompt condition (e.g., (32) and (34)) included a context sentence but no material in the second sentence prompt, and hence participants provide the entire second sentence. The distribution of first-mentioned referents allows us to estimate the next-mention bias *P*(referent), and the distribution of the forms of referring expression that participants use to mention these entities (pronoun or name) allows us to estimate the pronoun production bias *P*(pronoun|referent). These latter two quantities can be plugged into Eq (M1) to yield an estimated pronoun interpretation bias, which can then be compared with the actual interpretation bias measured in the Pronoun Prompt condition. They can also be used to compute predicted interpretation biases for the two models described in the next section.

#### Quantitative model evaluation

In addition to analyzing the results of our experiments in terms of their factorial designs, we present quantitative evaluations of the Bayesian model (M1) and two competing models. Each model makes predictions about the relationship among next-mention preferences *P*(referent), production biases *P*(pronoun|referent), and interpretation biases *P*(referent|pronoun) that can be evaluated on an item-by-item basis. Eq (M1) states the relationship predicted by the Bayesian model. The first competing model we refer to as the Expectancy model, according to which the comprehender’s interpretation bias toward a referent is (their estimate of) the probability that the referent is mentioned next in the context [8]. For this model, the predicted interpretation bias is estimated to be the next-mention bias *P*(referent). We express this model in (M2), using the ← assignment operator to emphasize the fact that this model does not follow normative probability theory.

$$\begin{array}{c} P(\text{referent}|\text{pronoun})\leftarrow P(\text{referent}) \end{array}$$ (M2)

The second competing model is what we call the Mirror model, according to which the interpretation bias toward a referent is proportional to the likelihood of the referent being pronominalized by the speaker. Once again, the assignment operator in Eq (M3) reflects the fact that this model does not follow normative probability theory.

$$\begin{array}{c} P\left(\text{referent}|\text{pronoun}\right)\leftarrow \frac{P(\text{pronoun}|\text{referent})}{\;\sum_{\text{referent}\in \text{referents}}\;P\left(\text{pronoun}|\text{referent}\right)} \end{array}$$ (M3)

This model captures the idea that comprehenders will assign pronouns based on their consideration of what entities the speaker is most likely to refer to using a pronoun instead of a competing referential form. This intuition is cached out by taking the comprehenders’ estimate of the probability that a speaker will produce a pronoun for a particular referent, normalized by the sum of the probabilities for all compatible referents.

In each experiment, we evaluate each of the above three models by three evaluation metrics. The first metric is the familiar R-squared (*R*^2^; previously used by [10]). *R*^2^ quantifies the strength of the linear relationship between the model predictions and observed behavior; for a model to perform well by *R*^2^, it must predict variability in item-by-item behavior that lines up well with the variability in the observed data. However, there are scenarios in which *R*^2^ can be high even if the model predictions are poorly calibrated—if the model systematically underestimates or overestimates the response variable, and/or the slope of the prediction/observation relationship is far from 1. The second and third metrics are Mean Squared Error (MSE) and Average Cross Entropy (ACE), with squared error and cross entropy defined respectively as follows:

$$\begin{array}{c} \text{Squared}\;\text{Error}={\left(p_{i}-{\hat{p}}_{i}\right)}^{2} \end{array}$$ (E1)

$$\begin{array}{c} \text{Cross}\;\text{Entropy}=p_{i}\;{\text{log}}_{2}\;{\hat{p}}_{i}+\left(1-p_{i}\right)\;{\text{log}}_{2}\;\left(1-{\hat{p}}_{i}\right) \end{array}$$ (E2)

where *p*_*i*_ and ${\hat{p}}_{i}$ are respectively the observed and predicted response proportions for the *i*-th item. Whereas for *R*^2^ higher values indicate better model performance, for MSE and ACE lower values indicate better performance, with perfect performance of MSE = ACE = 0 reached when $p=\hat{p}$ (i.e., all data points fall on the *x* = *y* line). Unlike *R*^2^, poorly calibrated model predictions necessarily lead to worse MSE and ACE. However, a model can sometimes achieve reasonably good MSE and ACE scores while capturing little of the observed variability in item-by-item predictions. The key difference between MSE and ACE for our purposes is that ACE, which can be thought of as an item-averaged model log-likelihood, more heavily weights absolute discrepancies between model predictions and observed behavior (as measured by $|p-\hat{p}|$) near the extreme values of $\hat{p}\approx 0$ and $\hat{p}\approx 1$, whereas MSE weights these absolute discrepancies equally throughout the entire range of $0\leq \hat{p}\leq 1$. Since each of these evaluation metrics emphasizes different features of a model’s predictions, the most complete picture of our three models’ relative performance will emerge from assessing these evaluation metrics together.

In order to determine model predictions in each experiment, we must estimate for each item the two probabilities *P*(referent) and *P*(pronoun|referent). We estimate these quantities from the Free Prompt conditions of our experiment. Our limited sample size intrinsically constrains the precision of our estimates. To help address this limited precision, and to avoid zero-probability estimates (which could lead to infinite cross-entropies or even to undefined model predictions for some items), we use a very basic form of additive smoothing, adding one pseudo-count to our item-specific experimental data for each logically possible combination of the *V* = 2 referents that might be re-mentioned (NP_1_ and NP_2_), and the *W* = 3 forms that might be used in a remention (null pronoun, the overt pronoun ‘ta’, name). This gives us item-specific probability estimates as follows:

$$\begin{array}{c} \hat{P}\left({\text{NP}}_{j}\right)=\frac{Count({\text{NP}}_{j})+W}{Count\left({\text{NP}}_{1}\right)+Count\left({\text{NP}}_{2}\right)+V\times W} \end{array}$$ (S1)

$$\begin{array}{c} \hat{P}\left(‘\text{ta}’|{\text{NP}}_{j}\right)=\frac{Count({\text{NP}}_{j}\wedge ‘\text{ta}’)+1}{Count({\text{NP}}_{j})+W} \end{array}$$ (S2)

Eq (S1) will thus be used to calculate the predictions of the Expectancy model (M2), Eq (S2) will be used to calculate the predictions of the Mirror model (M3), and both (S1) and (S2) will be used to calculate the predictions of the Bayesian model (M1).

## Experiment 1

The goal of Experiment 1 is to provide a baseline test case for investigating the generality of the Bayesian model as it applies to pronouns in Mandarin Chinese. Using the passage completion task with a 2-by-2 design, we seek to tease apart the respective influences of verb semantics and the grammatical roles of potential referents on pronoun interpretation.

### Methods

#### Ethics statement

This study (all three experiments included) was approved by the Human Research Protection Program, University of California San Diego (Project #141149). Participants completed the study through a web interface, which provided them with an informed consent form prior to beginning the study and indicated that initiating the study constituted their informed consent to participate.

#### Participants

We recruited 50 self-reported native Mandarin speakers (34 male and 16 female; age range: 18-47, mean age: 25.5, SD = 6.0) over Witmart, a China-based online crowd-sourcing platform [43]. The data were collected online between July and August 2015.

#### Materials and procedures

Participants carried out the passage completion task. Examples (32)–(35) illustrate the four conditions employed. Each participant completed 36 target items and 36 filler items with pseudo-randomization. Prompt Type varied within participants and within items; Verb Type varied only within participants. Each item was presented via a web interface on a separate page with a text box where the participants were instructed to write their continuations.

IC verbs are known to exhibit the strongest referential biases when the sentences participate in an Explanation coherence relation, that is, when the following sentence offers an explanation of the event described by the first sentence [13]. Therefore, to establish a robust baseline, in this experiment participants were explicitly asked to write a natural explanation for each prompt.

#### Coding

The responses were coded by two trained native speakers, the first author and a UC San Diego graduate student who was blind to the hypothesis of the study. Each judge went through the responses independently to code the first-mentioned referent (which, in the case of the Pronoun Prompt condition, is always the referent of the initial pronoun given in the prompt) in each continuation as belonging to one of five categories: *NP_1_, NP_2_, both, unclear* and *other*. If the sentence had embedded clauses, only the subject of the matrix clause was coded. If the two coders did not agree on the participant’s intended referent, or there was not enough information available to resolve the intended referent, the response was coded as *unclear*. For continuations in the Free Prompt condition, the speaker’s choice of referring expression was coded as *name, overt pronoun, null pronoun* or *other*.

### Results and discussion

Only continuations in which the first-mentioned entity was NP_1_ or NP_2_ were used for analysis; continuations were hence excluded if the next-mentioned referent was coded as *unclear* (3.8%), *both* (0.8%) or *other* (2.7%). Continuations were also excluded if the NP_1_ or NP_2_ mentions used a referring expression other than a name, an overt pronoun, or a null pronoun (1.0%). 8.3% of the total 1800 continuations were thus excluded, leaving a total of 1651 continuations for analysis.

All statistical analyses report results from mixed-effect logistic regression models with the maximal random-effect structure justified by the design [44], conducted using the lme4 R package. We report significance levels based on likelihood ratio tests. Predictors were sum-coded. In cases of convergence failure with lme4, we report analyses using the Markov Chain Monte Carlo (MCMC) methods in the R package MCMCglmm with *p*-values based on the posterior distribution of regression model parameters with an uninformative prior, as is common for MCMC-based mixed model fitting [45]. Error bars in all figures are standard errors over by-participant means.

The weak form of the Bayesian hypothesis predicts that pronoun interpretation biases and production biases follow Bayesian principles, without fully specifying what factors affect each component. The strong form of the hypothesis additionally predicts that semantic factors (Verb Type) affect only next-mention biases *P*(referent) and not pronoun production biases *P*(pronoun|referent). The primary factor affecting pronoun production is predicted to be the grammatical role of the referent (Re-mentioned NP). We test these predictions in the following analyses.

#### Next-mention biases

**Fig 1:**
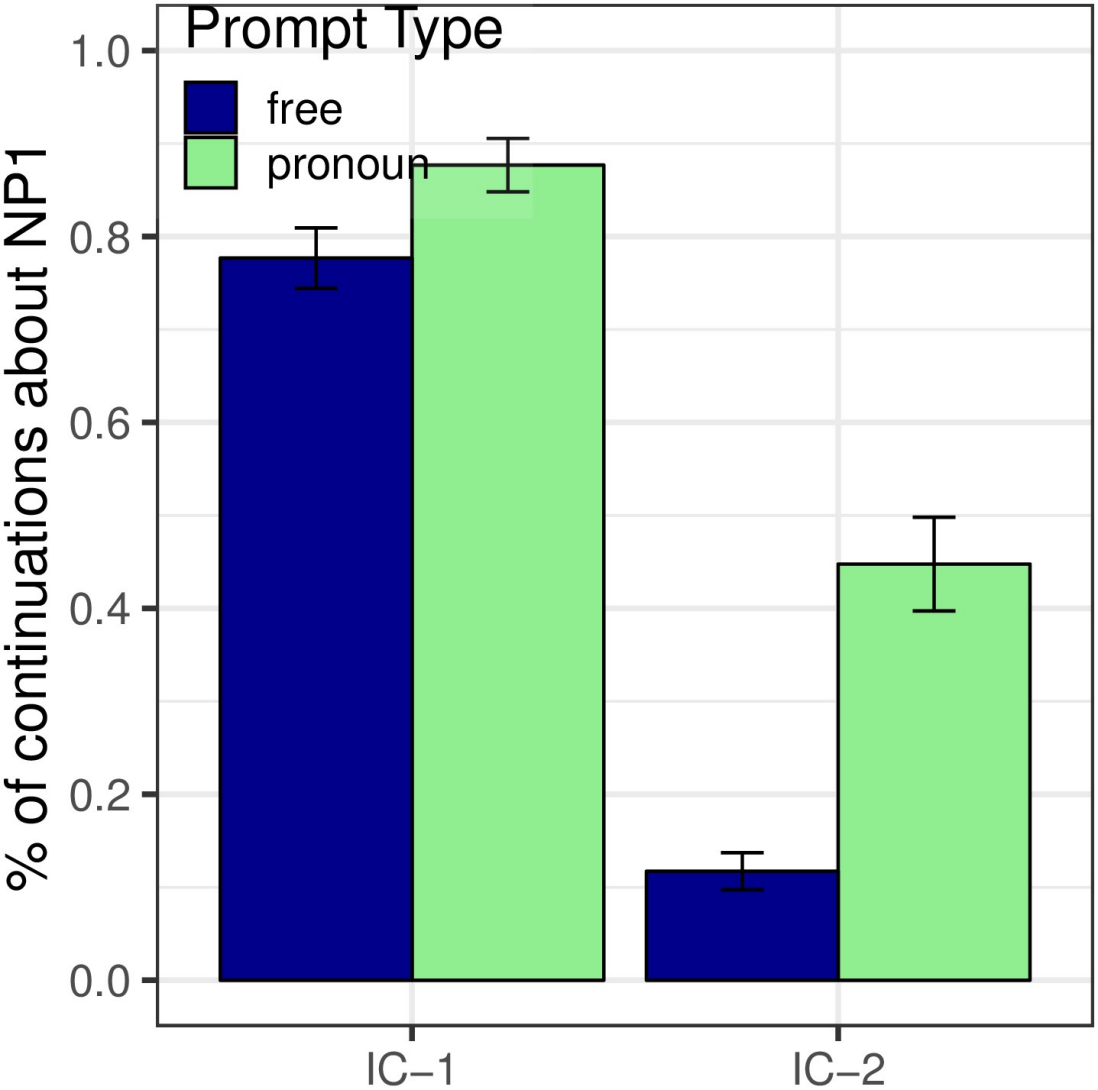
Expt 1: Proportion of continuations about the subject.

Fig 1 shows the proportion of continuations about NP_1_ in both Free Prompt and Pronoun Prompt conditions. We first evaluate the next-mention biases (dark blue bars) in the Free Prompt data, which serves to estimate the prior in the Bayesian model. Analyses showed a main effect of Verb Type (*p* < 0.001): subject-biased IC-1 items prompted significantly more continuations about NP_1_ (77.7%) than object-biased IC-2 items (11.7%). Verb semantics therefore had a strong effect on the next-mention biases, which is to be expected given that these verbs were selected according to this criterion after the norming study.

#### Production biases

**Fig 2:**
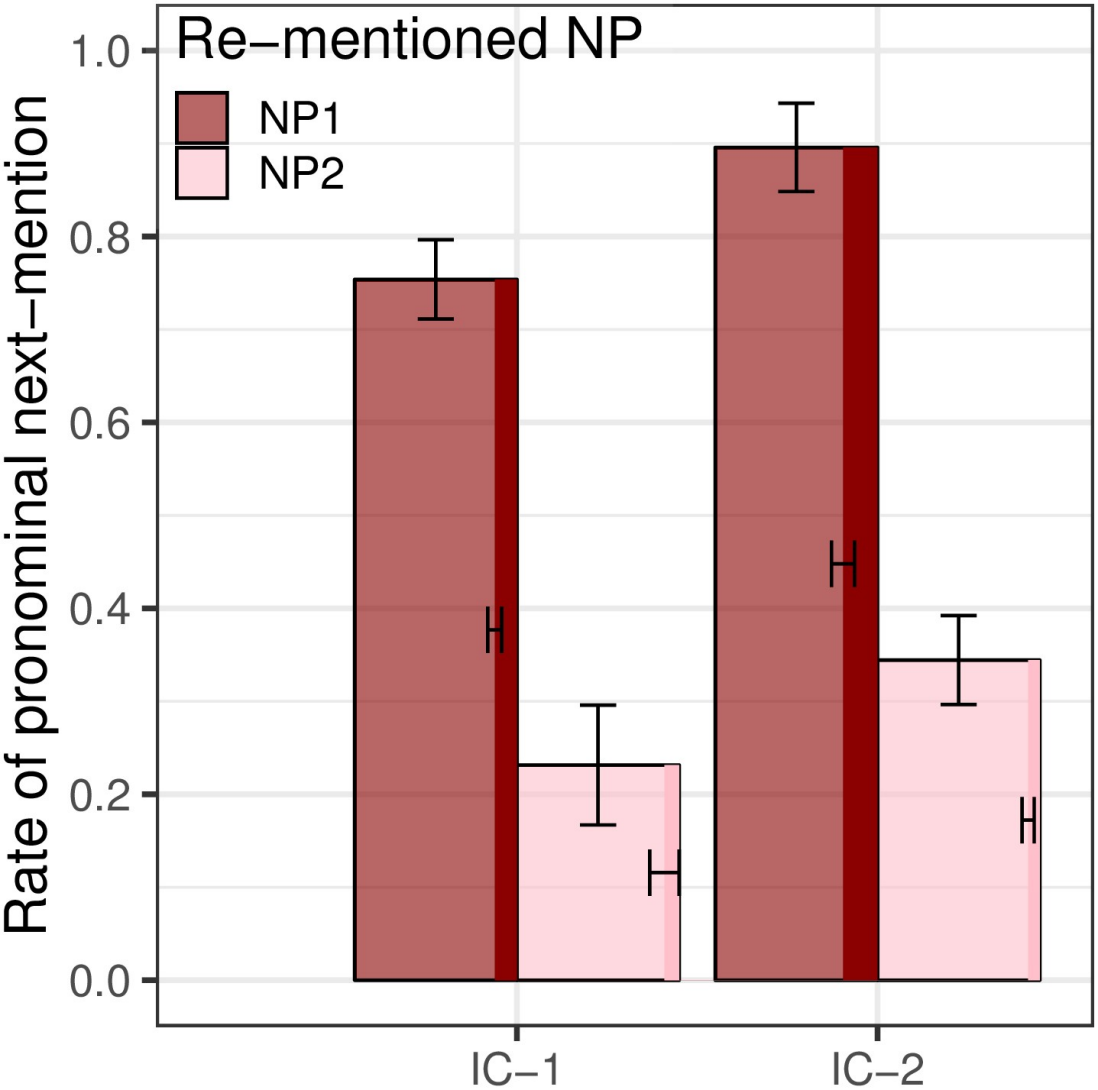
Expt 1: Rate of pronominalization, by Verb Type and Re-mentioned NP.

Fig 2 shows rates of pronominalization conditioned on the Re-mentioned NP when null and overt pronoun continuations are collapsed. Because our pronoun prompt conditions only utilized overt pronouns (due to the inherent difficulty of signaling the existence of a null pronoun in a prompt), we want to ensure that the two types of pronouns are used in similar ways. The distribution of the two types of pronoun was similar across referents for each verb type, with the overt pronoun *ta* being the majority choice for each condition. Moving from left to right in Fig 2, the percentages of overt pronouns among both overt and null pronouns used were 86%, 90%, 79%, and 93%.

Analyses revealed a large main effect of Re-mentioned NP (*p*_*MCMC*_ < 0.001), with NP_1_ re-mentions significantly more likely to be pronominalized than NP_2_ re-mentions. However, there was no significant interaction between Re-mentioned NP and Verb Type (*p*_*MCMC*_ = 0.524), indicating that the verb-semantic factors that strongly affect next-mention biases do not influence a speaker’s decision about whether to use a pronoun. These results align with the predictions of both the strong and weak hypotheses, indicating a clear disassociation between next-mention biases and production biases. There was also a smaller, unanticipated main effect of Verb Type (*p*_*MCMC*_ = 0.011), with pronominalization rates higher in IC-2 contexts than in IC-1 contexts. The reasons for this main effect remain unclear.

#### Interpretation biases

Now we examine the interpretation biases measured in the Pronoun Prompt condition (the posterior, depicted in the green bars of Fig 1) and compare them to next-mention biases (the prior, already seen in the blue bars of Fig 1). First, the Bayesian model predicts a monotonic positive relationship between prior and posterior: IC-1 items should elicit more NP_1_ interpretations than IC-2 items in the pronoun condition. This prediction is confirmed: a significant effect of verb type was found within the Pronoun Prompt condition (*p* < 0.001), in the same direction as the effect found in the Free Prompt condition. Second, the Bayesian model predicts an effect of likelihood on the posterior, specifically skewing references toward the subject as compared to the next-mention biases. We see this result in Fig 1 as a significant main effect of Prompt Type (*p* < 0.001), with more NP_1_ re-mentions in the Pronoun Prompt condition than in the Free Prompt condition.

Analyses also showed an interaction between Prompt Type and Verb Type (*p* < 0.01), whereby the effect of Pronoun prompt was larger for IC-2 verbs than IC-1 verbs. The interaction’s presence was neither predicted nor precluded.

#### Quantitative model comparisons

We conduct quantitative comparisons among the Bayesian, Expectancy, and Mirror models by computing their item-by-item predictions for interpretation preferences of the overt pronoun *ta*, *P*(referent = NP_1_|*ta*), and comparing them against item-by-item behavior in the Pronoun Prompt condition.

**Fig 3:**
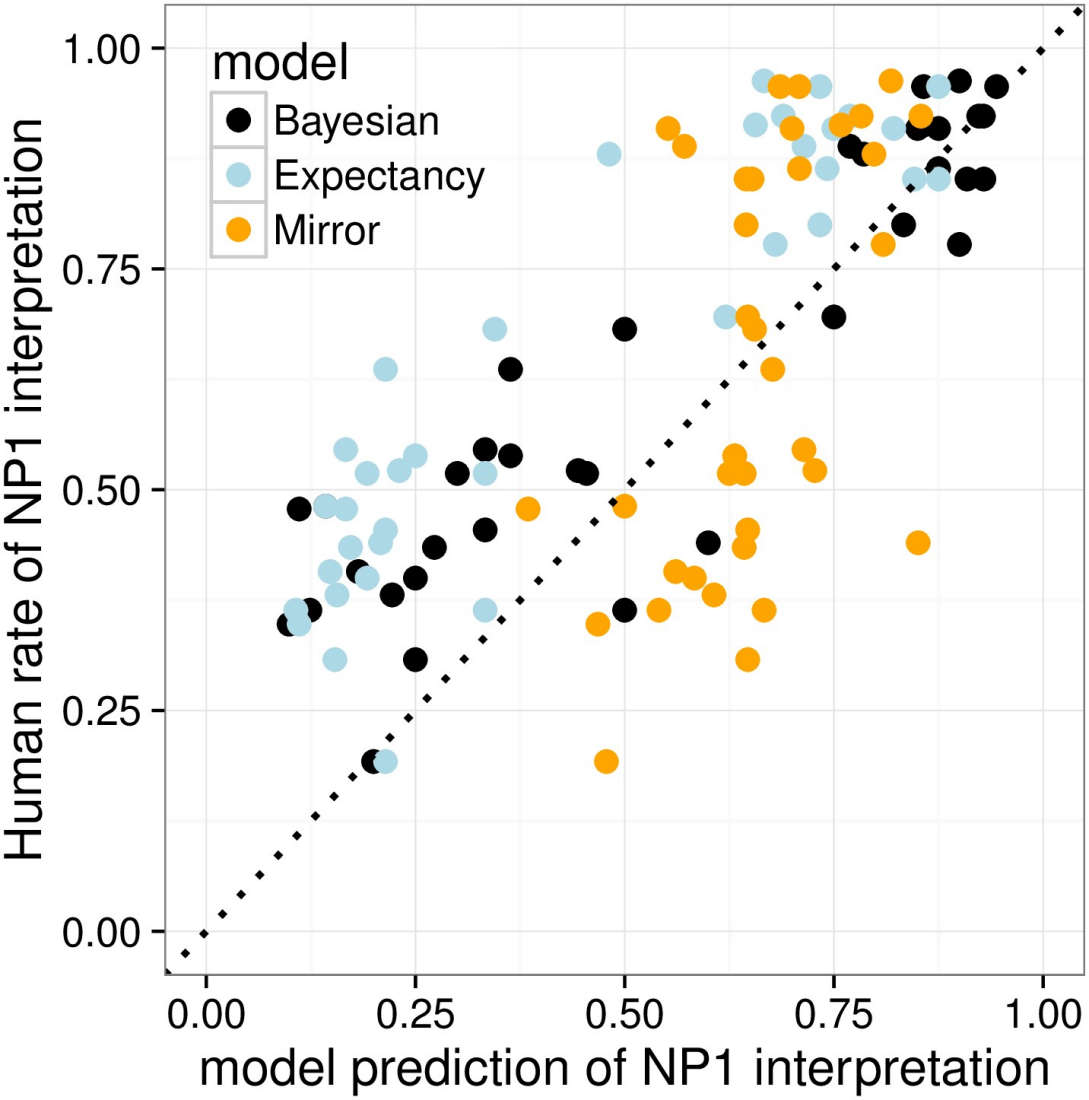
Expt 1: Item-by-item quantitative model evaluation for Experiment 1.

Fig 3 plots observed NP_1_ interpretation rates against item-specific predictions of the three models. The *x* = *y* dotted line would be a perfect model fit. Table 1 shows the performance of each model on each of the three evaluation criteria: *R*^2^, MSE, and ACE. The Bayesian model has the best performance according to all three measurements: it accurately predicts cross-item variability in interpretive preferences across items, and is generally well calibrated, though there are signs that it slightly underpredicts NP_1_ interpretation rates on the left side of the graph (these are IC- 1 items). The Mirror model severely underpredicts cross-item variability in interpretation preference, because it lacks the influence of the prior, so it performs particularly badly on *R*^2^. The Expectancy model does a good job predicting cross-item variability and so does well on *R*^2^, but it systematically and substantially underpredicts the rate of NP_1_ pronoun interpretation because it lacks the likelihood-derived bias toward NP_1_ obtaining from the speaker’s choice of pronominal form, leading to the worst performance on MSE and ACE.

**Table 1:** Expt 1: R-squared, MSE, and ACE of the three models.

	Bayesian	Expectancy	Mirror
*R*^2^	**0.840**	0.825	0.278
*MSE*	**0.024**	0.055	0.040
*ACE*	**0.852**	0.967	0.896

MSE = Mean squared error; ACE = Average cross entropy.

To further evaluate the models, we performed paired t-tests on by-item squared error and cross-entropy (see Eqs (E1) and (E2)) between the Bayesian model and the other two models. (*R*^2^ measures the fit of coefficients and cannot be evaluated using paired t-tests.) For MSE, the Bayesian model performed significantly better than the Expectancy model (*p* < 0.001) but not the Mirror model (*p* = 0.119). For ACE, the Bayesian model significantly outperformed both the Expectancy model (*p* < 0.001) but not the Mirror model (*p* = 0.242).

### Summary

The results from Experiment 1 show that verb semantics had a strong effect on next-mention biases, whereby IC-1 verbs elicited more mentions of the subject than IC-2 verbs did. They also showed that grammatical roles of potential referents had a significant impact on the likelihood of producing a pronoun given a particular referent, with re-mentions of the subject of the previous sentence (NP_1_) more likely to be pronominalized than re-mentions of the non-subject (NP_2_). The lack of an interaction further revealed that, as predicted by the strong Bayesian model, semantic factors do not influence pronoun production biases, even though comprehenders leverage those factors in making an interpretation decision.

These results provide evidence that when interpreting an ambiguous pronoun, Mandarin speakers take into account both the prior probability of a referent being mentioned next in the context and the likelihood that the speaker would have chosen to use a pronoun to mention that referent, lending support to both the strong and the weak versions of the Bayesian hypothesis.

## Experiment 2

Experiment 2 further tests the generality of the Bayesian theory by pursuing a set of findings regarding the effect of voice on pronoun behavior identified for English by [10]. Recall that Rohde & Kehler hypothesized that a difference in syntactic form—specifically active vs. passive voice—would have an effect on production biases, on the hypothesis that it is topichood, rather than grammatical role, that drives production biases (henceforth, the Topichood Hypothesis).

Hypothesizing that being the subject of a passive clause is a stronger indicator of topichood than being the subject of an active clause, Rohde & Kehler thus predicted that the syntactic subject of a passive clause is more likely to be pronominalized than that of an active clause. The results of their study confirmed this prediction. However, the results also revealed a separate effect, unexpected under the strong hypothesis, whereby passivization affected the next-mention bias as well. Counterintuitively, this effect went in the opposite direction of what one might expect: it increased the next-mention rate of the logical subject, that is, the entity mentioned from within a *by*-adjunct.

Here we evaluate the predictions of the models by employing a similar voice manipulation in Mandarin Chinese. Following [10], the Topichood Hypothesis predicts that the difference in information structure between active and passive voice will affect production biases for referents in subject position. Likewise, recall that the *bei* construction places a constraint requiring that the logical object be affected; in so far as the construction more strongly enforces a construal in which NP_1_ is viewed as affected as compared to the canonical active construction, the next-mention bias for the logical object might be increased as well. Since affectedness is a semantic property, such an effect is neither predicted nor precluded on the strong hypothesis. Further, the weak hypothesis makes no commitment to the either of these predictions; instead, it simply predicts that any change in the pronoun production biases or the next-mention biases would affect interpretation biases, in accordance with Bayes’ rule.

### Methods

#### Participants

We recruited 71 self-reported native Mandarin speakers (41 male and 30 female; age range: 18-38; mean age: 24.6; SD: 4.3) over Witmart. None of them participated in any other studies reported in this paper. The data were collected online between August and September 2015.

#### Materials and procedures

The same passage completion paradigm as Experiment 1 was employed. In addition, we included a Voice (active vs. passive) manipulation. Participants were asked to write a natural continuation given the prompt. Since the change of information structure in the prompt sentence might have potential effects on next-mention bias and/or coherence relations, participants were not explicitly instructed to write explanations as was done in Experiment 1. The verbs were identical with the ones in Experiment 1 with one exception: *anwei* (‘comfort’) was replaced with *xiaokan* (‘look down upon’) since the former sounds unnatural in a passive clause. Example stimuli in passive voice are shown in (36)–(39). For stimuli in active voice, see (32)–(35).

(36) [${}_{{\text{NP}}_{1}}$ 洁怡] 被 [${}_{{\text{NP}}_{2}}$ 美惠] 打动了_IC-1_. _________ [IC-1, Free]
[${}_{{\text{NP}}_{1}}$ Jieyi] bei [${}_{{\text{NP}}_{2}}$ Meihui] dadong-le_IC-1_. _________
Jieyi was_by Meihui touched. _________
(37) [${}_{{\text{NP}}_{1}}$ 洁怡] 被 [${}_{{\text{NP}}_{2}}$ 美惠] 打动了_IC-1_. 她 _________ [IC-1, Pronoun]
[${}_{{\text{NP}}_{1}}$ Jieyi] bei [${}_{{\text{NP}}_{2}}$ Meihui] dadong-le_IC-1_. Ta _________
Jieyi was_by Meihui touched. She _________
(38) [${}_{{\text{NP}}_{1}}$ 洁怡] 被 [${}_{{\text{NP}}_{2}}$ 美惠] 打动了_IC-2_. _________ [IC-2, Free]
[${}_{{\text{NP}}_{1}}$ Jieyi] bei [${}_{{\text{NP}}_{2}}$ Meihui] jiegu-le_IC-2_. _________
Jieyi was_by Meihui fired. _________
(39) [${}_{{\text{NP}}_{1}}$ 洁怡] 被 [${}_{{\text{NP}}_{2}}$ 美惠] 打动了_IC-2_. 她 _________ [IC-2, Pronoun]
[${}_{{\text{NP}}_{1}}$ Jieyi] bei [${}_{{\text{NP}}_{2}}$ Meihui] jiegu-le_IC-2_. Ta _________
Jieyi was_by Meihui fired. She _________

Participants wrote passage continuations using the same web interface that was used in Experiment 1.

#### Coding

The two trained judges used in Experiment 1 coded the responses for next-mention referent and choice of referring expression using the same criteria as those in Experiment 1. Each continuation was also coded for its coherence relation, with categories *Explanation, Result, Elaboration*, and *Occasion*—as well as *Other*.

### Results and discussion

Out of 2556 continuations, 8.7% were excluded because the next-mentioned referent was coded as *unclear* (3.8%), *both* (2.6%) or *other* (2.0%), or the choice of referring expression was other than a name, an overt pronoun, or a null pronoun (0.3%). This left a total of 2334 continuations in the dataset.

#### Next-mention biases

**Fig 4:**
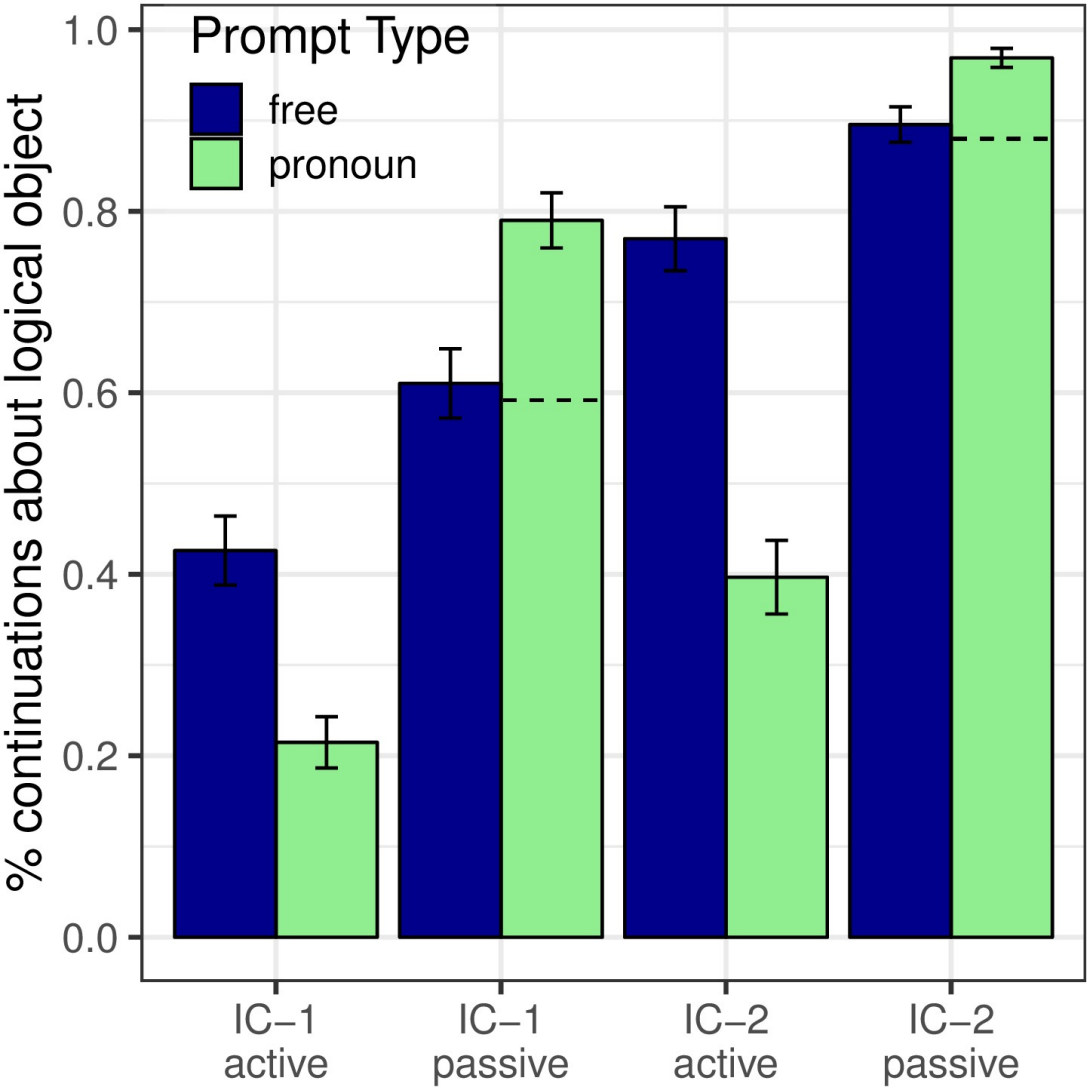
Expt 2: Proportion of continuations about the logical object. Dashed lines illustrate the NP_1_ next-mention rate that would be predicted for passives under an alternative model that is identical to the Bayesian model except that the prior is replaced with the active-voice prior.

Next-mention results are depicted in Fig 4 (blue bars) in terms of the proportion of continuations about the logical object; this visualization clearly illustrates the effect of the voice manipulation. The Free Prompt condition (blue bars) showed a main effect of Verb Type (*p* < 0.001), whereby IC-2 verbs elicited more continuations about the logical object than IC-1 verbs. The effect of Verb Type is compatible with both the strong and the weak versions of the Bayesian hypothesis.

Analyses also revealed a main effect of Voice (*p* < 0.001) in the Free Prompt condition, whereby passivization led to an increase in continuations about the logical object. There was no significant interaction between Verb Type and Voice (*p* = 0.597). These results are compatible with both the strong and weak Bayesian hypotheses as well.

**Fig 5:**
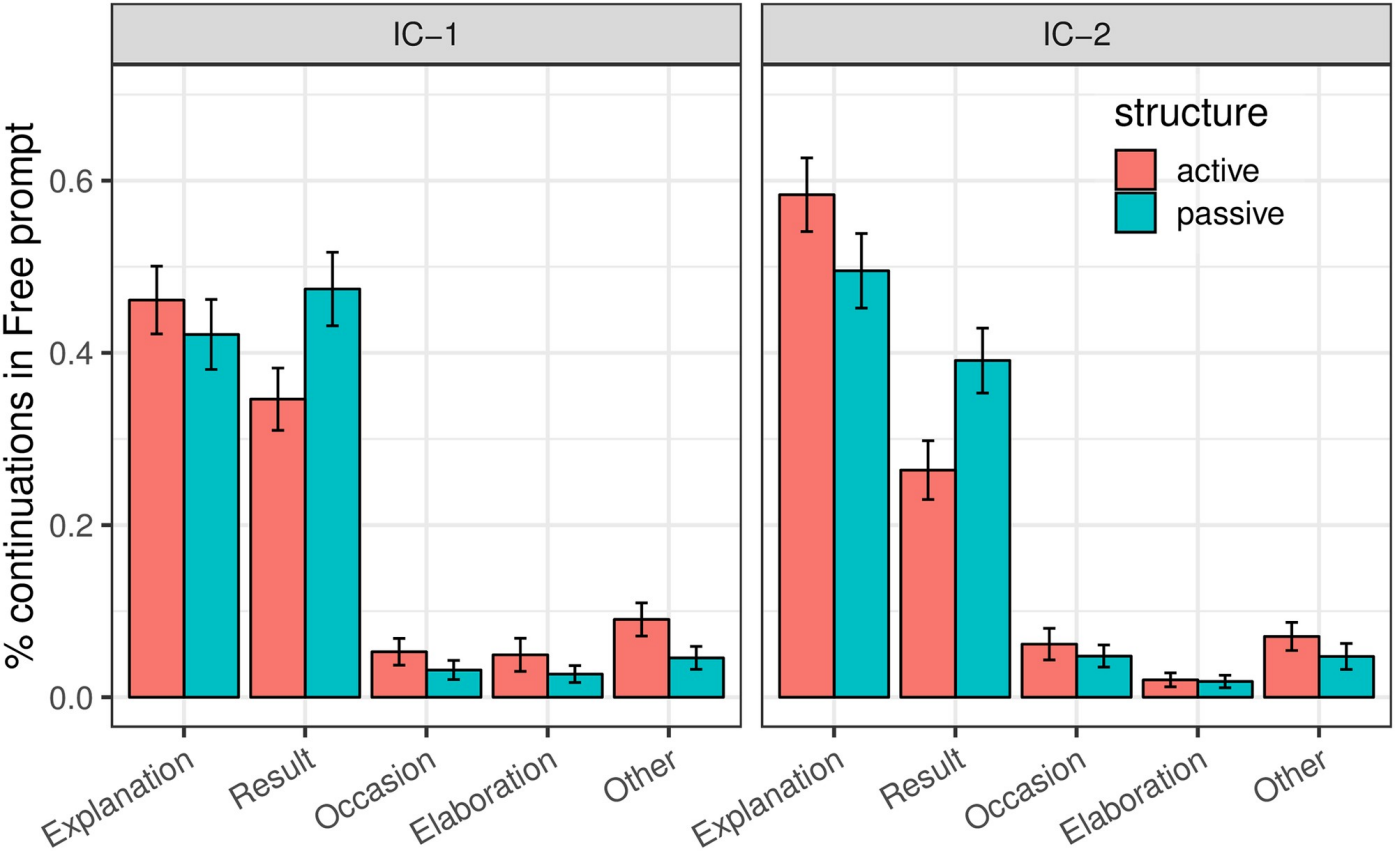
Expt 2: Proportion of continuations by coherence relations in Free Prompt condition.

We hypothesized that the passive’s imputing affectedness to the logical object might have an effect on continuation preferences: greater affectedness of the logical object might encourage Result continuations that describe how the logical object was affected. Analysis of the distribution of coherence relations in different experimental conditions, and of re-mention rates within each coherence relation, are consistent with this hypothesis. Fig 5 shows the distribution of coherence relations in each experimental condition. Passivization elicited more Result continuations, which are associated with a next-mention bias toward the logical object in both IC-1 and IC-2 contexts [15, 46, 47]. (We only include Explanations and Results in all coherence relation analyses due to the relative infrequency of other coherence relations.) A mixed logit analysis on the proportion of Result coherence relations relative to Explanation relations with Voice, Verb Type and their interaction as predictors finds a main effect of Voice (*p* = 0.009), whereby passive sentences had more Result continuations than active sentences, and a small effect of Verb Type (*p* = 0.011), with more Result continuations for IC-1 verbs than for IC-2 verbs, and no interaction (*p* = 0.295).

**Fig 6:**
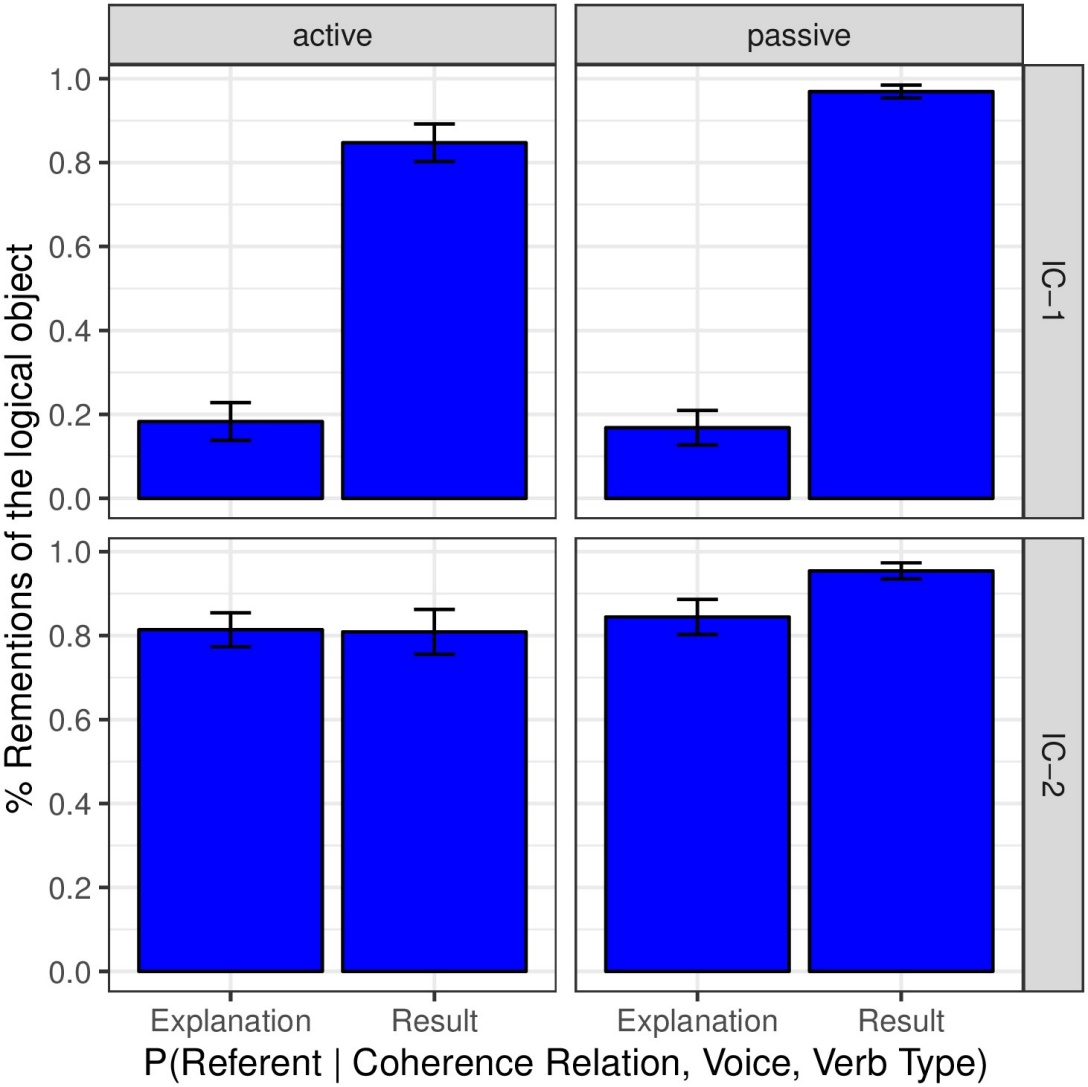
Expt 2: Next-mention biases in explanation and result coherence relations by Verb Type and by syntactic construction.

**Fig 7:**
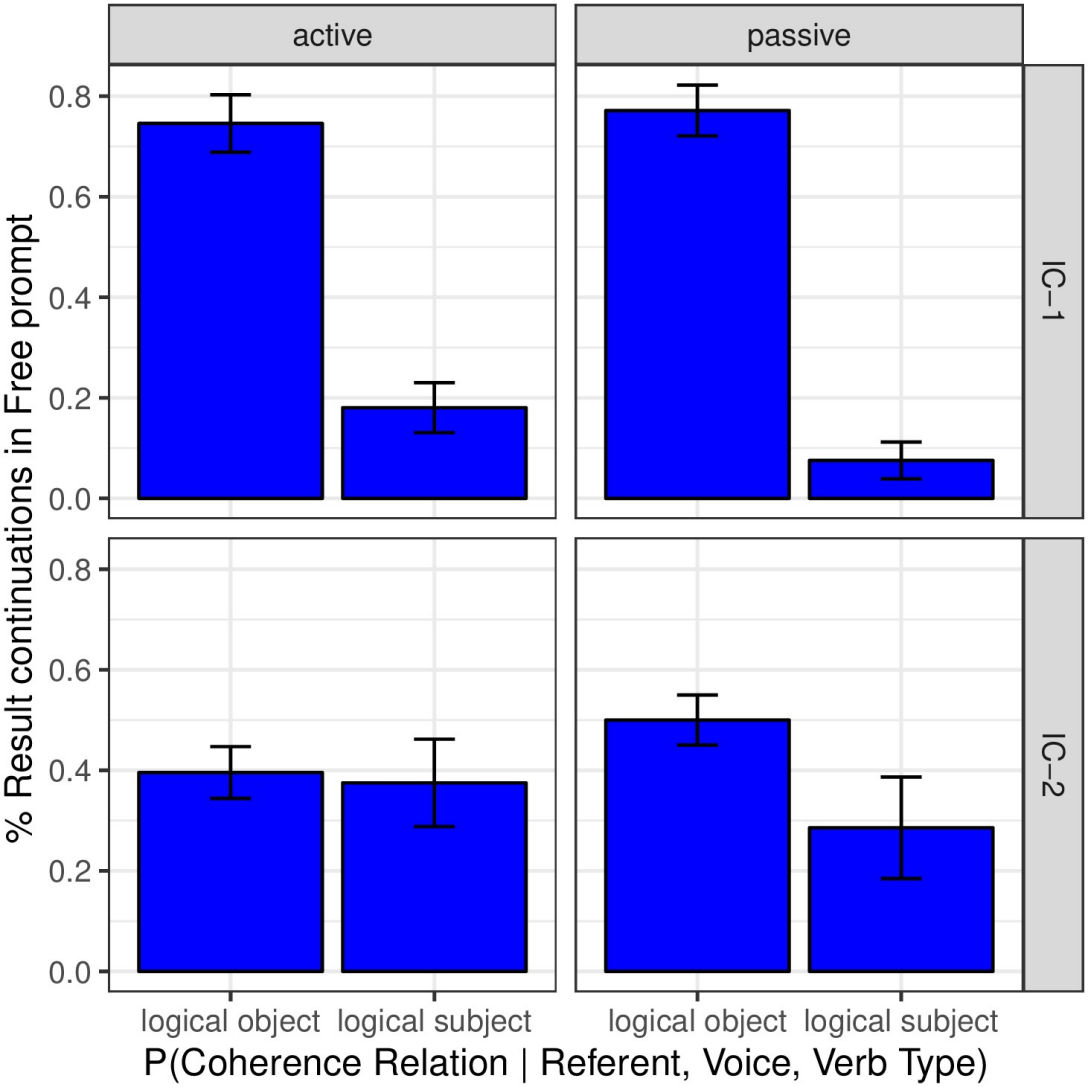
Expt 2: Coherence relations by Verb Type, syntactic construction, and next mention.

Whereas the results provide evidence for our hypothesis concerning the role of voice on coherence and next mention, we should be clear that they do not suffice to reveal the full structure of the causal dependencies that relate these three factors. To further investigate this question, we examine to what extent the conditional distribution of each of the two outcomes (coherence and next mention) on the other varies depending on grammatical voice. If the conditional distribution of next mention given coherence relation varies little with grammatical voice, but the conditional distribution of coherence relation given next mention varies substantially with grammatical voice, it would provide supporting evidence that coherence relation plays a crucial mediating role. In turn, the opposite pattern would provide supporting evidence for a crucial mediating role of next mention. The two sets of conditional distributions can be seen in Figs 6 and 7, where only completions that participate in Explanation and Result relations are analyzed. Evidence for the mediating role of coherence on next mention can be seen in Fig 7, in which the rate of Result relations is significantly lower for logical subjects in the passive than in the active for IC- 1 verbs, and is significantly higher for logical objects in the passive than in the active for IC- 2 verbs (both *p* < 0.01). Fig 5 provides another view of the evidence for the mediating role of coherence on next mention. If the relationship between voice and coherence was completely mediated by next mention, one would expect passivization in IC-2 contexts to result in a proportionally greater number of both Explanation and Result relations as compared to active contexts, since both relations are strongly object-biased in IC-2 contexts. However, whereas the distribution of Result relations revealed the expected increase, the percentage of Explanation relations actually decreased. This pattern thus offers support for the existence of a direct dependency between voice and coherence in the predicted manner. On the other hand, some small, but nonetheless statistically reliable, pairwise differences can be seen in Fig 6 as well: for both verb types the rate of logical object re-mention when a Result relation is operative is reliably higher in the passive than in the active (*p*_*MCMC*_ = 0.031 for IC- 1 and *p*_*MCMC*_ = 0.018 for IC- 2 respectively). As an ensemble, the data thus do not strongly adjudicate among hypotheses regarding the structure by which the two outcomes mediate one other. Future investigation may provide further insights.

#### Production biases

**Fig 8:**
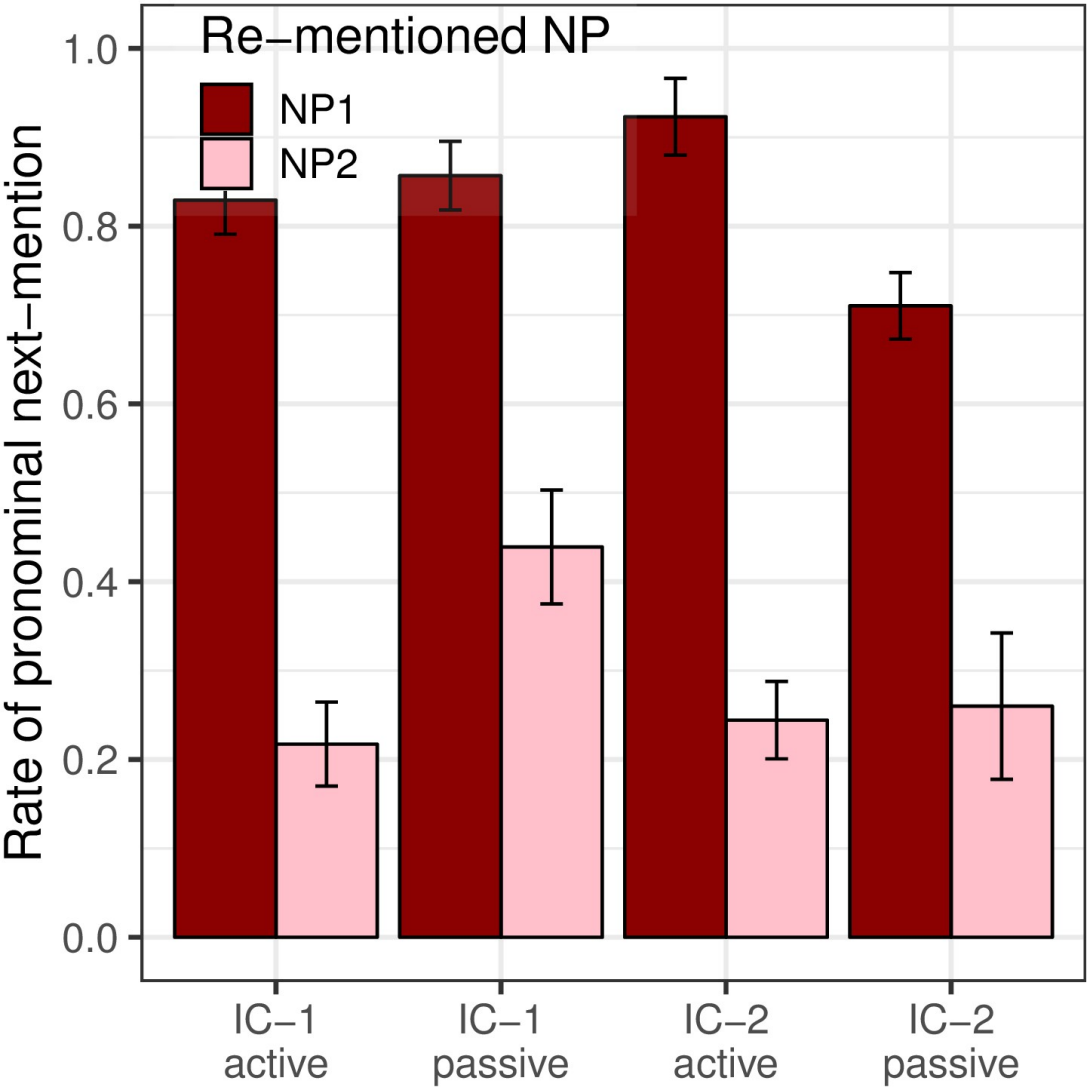
Expt 2: Rate of pronominalization the the free prompt data (Collapsing null and overt pronouns).

Fig 8 presents the rate of pronominalization including both null and overt pronouns. Results showed a main effect of Re-mentioned NP (*p*_*MCMC*_ < 0.001), with NP_1_ more likely to be pronominalized than NP_2_. There was no main effect of Voice (*p*_*MCMC*_ = 0.64) or Verb Type (*p*_*MCMC*_ = 0.90). The higher rate of pronominalization of NP_1_ and the lack of effect of Verb Type are both predicted by the strong version of the Bayesian hypothesis. The lack of an effect of Voice, however, runs counter to the predictions of the Topichood Hypothesis, on the assumption that the syntactic subject of a passive clause in Mandarin is more likely to be the sentence topic than the subject of an active clause. These results instead suggest that grammatical role (the surface syntactic position) is the primary factor affecting pronoun production biases. Additionally, there was a Re-mentioned NP × Voice interaction (*p*_*MCMC*_ = 0.002) and a Voice × Verb Type interaction (*p*_*MCMC*_ = 0.002). The three-way interaction (Re-mentioned NP × Verb Type × Voice) was significant (*p*_*MCMC*_ = 0.038), but the effect is small and the reason remains unclear.

#### Interpretation biases

**Fig 9:**
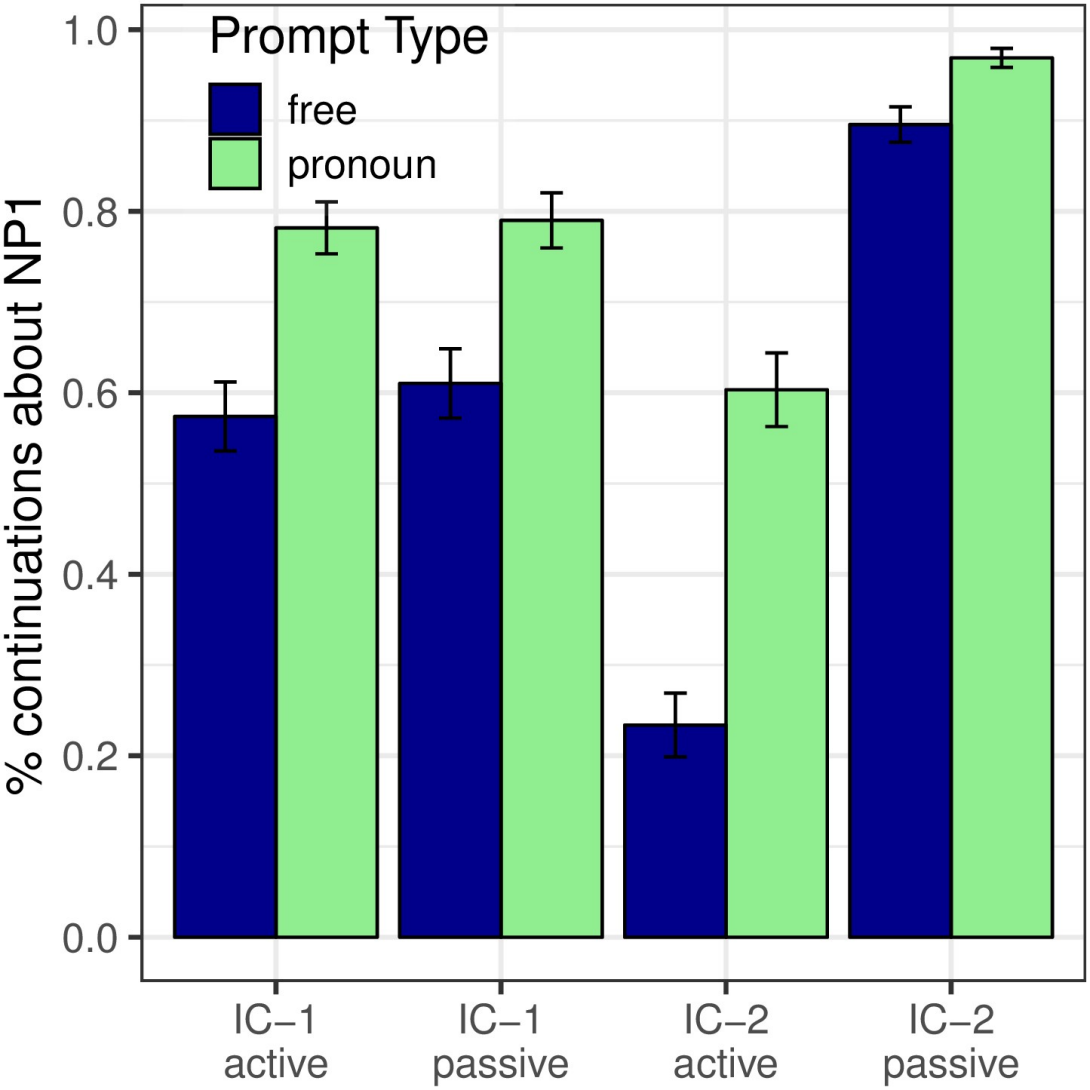
Expt 2: Proportion of continuations about the syntactic subject by Verb Type and Prompt Type. The data in this figure are identical to those in Fig 4, but are plotted here in terms of proportion of continuations to NP_1_, to facilitate visualization of the effect of the likelihood.

As with Experiment 1, we test two key predictions shared by both versions of the Bayesian model. The first is that the effects of verb type and passivization on next-mention preferences that are seen in the Free Prompt condition (Fig 9, blue bars) should also be seen in the Pronoun Prompt condition (Fig 4, green bars). Our data confirmed this prediction: These main effects of Verb Type (*p* < 0.001) and Voice (*p* < 0.001) in the Pronoun Prompt condition both match the corresponding effects seen in the Free Prompt condition. However, there is a potential limitation to this conclusion with respect to the effect of Voice on interpretation biases that we will address momentarily.

The second key prediction is the effect of the likelihood term in Eq (M1): the Pronoun Prompt condition should elicit more re-mentions of NP_1_ than the Free Prompt condition. This prediction is also borne out in our data, as can be seen most clearly by re-plotting the data from Fig 4 so that the y-axis shows the proportion of continuations about NP_1_, which we present in Fig 9. Analyses showed a main effect of Prompt Type (*p*_*MCMC*_ < 0.001), with Pronoun prompts being associated with more NP_1_ re-mentions than Free prompts.

Analyses also revealed a main effect of Voice (*p*_*MCMC*_ < 0.001), with passive voice eliciting more NP_1_ re-mentions than active voice. There was a small effect of Verb Type (*p*_*MCMC*_ = 0.038), where IC-1 verbs elicited more NP_1_ re-mentions overall. Additionally, there was a significant interaction between Voice and Verb Type (*p*_*MCMC*_ < 0.001), with IC-2 verbs more sensitive to the change of Voice with respect to re-mentions. None of these results are theoretically crucial.

We now address the remaining limitation regarding the potential effect of voice on interpretation bases. In Fig 4 (green bars), one would ideally like to conclude from the main effect of Voice that the effect of passivization on the next-mention prior—an increased bias toward the logical object—is also reflected in interpretive preferences. In the Pronoun Prompt condition, however, under the Bayesian model this effect is confounded with the effect of the likelihood, because passivization shifts the logical object to the subject position, which in turn increases the likelihood that a re-mention of the logical object would take the form of a pronoun. We address this potential confound by examining the predictions of an alternative hypothetical model which is identical to the Bayesian model of Eq (M1), except that the normatively correct prior term *P*(referent = NP_1_|passive) is replaced with the active-voice prior term *P*(referent = NP_1_|active). We depict the predictions of this model with dashed lines on the green bars of Fig 4. This alternative model underpredicts the observed rate of logical object re-mention, consistent with an interpretation where the increase in NP_1_ pronoun interpretation from the passive voice is partly due to its effect on next-mention biases, and not just to its effect on production biases. Collectively, then, the results of this experiment support all the key predictions of the Bayesian model.

#### Quantitative model comparisons

**Fig 10:**
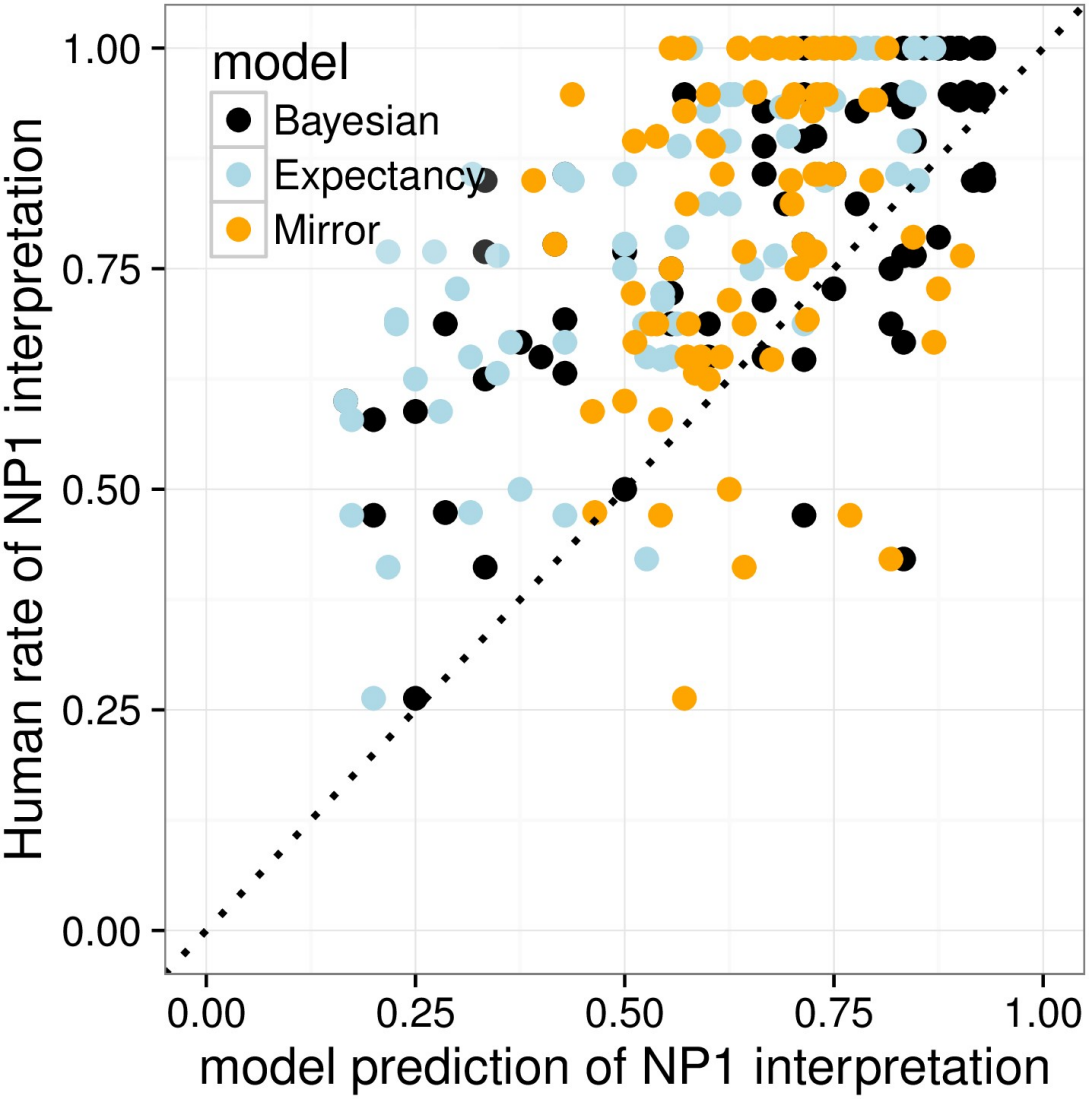
Expt 2: Item-by-item quantitative model evaluation for Experiment 2.

Fig 10 and Table 2 show the performance of the three models. Similar to the patterns seen in Experiment 1, the Mirror model severely underpredicts cross-item variability in interpretation preferences, and the Expectancy model systematically underestimates the likelihood-derived preference toward NP_1_. The Bayesian model performed the best on MSE and ACE, and had the second best R-squared value; the Expectancy model had the highest *R*^2^ but the worst MSE and ACE. We performed paired t-tests on by-item squared error and cross-entropy (see Eqs (E1) and (E2)) between the Bayesian model and the other two models. For MSE, the Bayesian model performed significantly better than the Expectancy model (*p* < 0.001) but not the Mirror model (*p* = 0.137). For ACE, the Bayesian model significantly outperformed both the Expectancy model (*p* < 0.001) and the Mirror model (*p* = 0.044).

**Table 2:** Expt 2: R-squared, MSE, and ACE of the three models.

	Bayesian	Expectancy	Mirror
*R*^2^	0.465	**0.569**	0.043
*MSE*	**0.043**	0.077	0.055
*ACE*	**0.758**	0.881	0.828

MSE = Mean squared error; ACE = Average cross entropy.

We conjecture that the Expectancy model achieves an *R*^2^ superior to the Bayesian model because its predictions involve only one noisy estimate, $\hat{P}\left(\text{referent}\right)$, whereas the Bayesian model involves the product of two noisy estimates, the prior $\hat{P}\left(\text{referent}\right)$ and the likelihood $\hat{P}\left(\text{pronoun}|\text{referent}\right)$. Because the Expectancy model omits the likelihood, it underpredicts NP_1_ interpretations more severely than the Bayesian model. While this across-the-board underprediction yields poor MSE and ACE for the Expectancy model, it does not hurt its *R*^2^, which is not sensitive to overall model calibration.

### Summary

The results from Experiment 2 followed those of Experiment 1 in confirming several fundamental predictions of the model. First, verb biases affected both next-mention biases and pronoun interpretation biases, but not pronoun production biases, as predicted by the strong form of the Bayesian model. Second, production biases were driven by the grammatical role of the referent. Finally, the production bias also influenced interpretation biases, such that continuations after pronoun prompts resulted in a greater number of next mentions of the subject than those following free prompts.

Unlike what Rohde and Kehler [10] found for the passive voice in English, we found that the passive *bei* construction increases the rate of logical object re-mention compared to the canonical active in Mandarin. Our analysis of next-mention biases in Experiment 2 suggests that coherence relations play at least a partial role in mediating this effect: passive *bei* elicits more Result continuations than the canonical active voice, and Result continuations tend to be about the logical object. Affectedness has been proposed to be a meaning component of the Mandarin passive that is less prominent or even absent in the English passive [25, 26]; our results suggest that the presence or absence of such meaning components across constructions and languages may have effects on pronoun interpretation through their effects on next-mention preferences and principles of Bayesian inference.

Also running counter to Rohde and Kehler [10] was the lack of consistent evidence that rementions of passive subjects are pronominalized more often than rementions of canonical active subjects. Whereas their predictions were borne out for English, we found no similar effect for Mandarin. Instead, the results offer provisional support for the idea that grammatical role is the primary determinant of production biases.

## Experiment 3

The results of Experiment 2 and those of Rohde and Kehler on English [10] provide inconsistent evidence on the question of whether pronoun production biases are conditioned primarily on topichood or on grammatical role. As discussed previously, it is perhaps difficult to tease apart the respective influences of grammatical role and topichood on pronoun production, given the high degree of correlation between the two. If the *ba*-active construction in Mandarin Chinese marks topichood, however Tsao [27], may allow us to see an effect of topichood independent of grammatical role. Exploring this possibility is the primary goal of Experiment 3. If the *ba*-active construction raises the level of topichood of the direct object as compared to the canonical active, the Topichood Hypothesis predicts that the *ba*-object will be pronominalized more often than its non-*ba* counterpart. The Bayesian models further predict that any change in the pronoun production or next mention biases that result from the syntactic manipulation will likewise affect interpretation biases in the manner specified by Bayes’ Rule.

### Methods

#### Participants

We recruited 87 self-reported native Mandarin speakers (45 male and 42 female; age range: 18-46; mean age: 27.1; SD: 5.1) over Witmart. None of them participated in any other studies reported in this paper. The data were collected online in April 2016.

#### Materials and procedures

The same passage completion paradigm as described in Experiment 2 was employed. Instead of Voice manipulation, a Syntactic Construction (canonical active vs. *ba*-active) manipulation was included. Example stimuli in *ba* constructions are shown in (40)–(43). For stimuli in active canonical sentences, see (32)–(35).

(40) [${}_{{\text{NP}}_{1}}$ 美惠] 把 [${}_{{\text{NP}}_{2}}$ 洁怡] 打动了_IC-1_. _________ [IC-1, Free]
[${}_{{\text{NP}}_{1}}$ Meihui] bei [${}_{{\text{NP}}_{2}}$ Jieyi] dadong-le_IC-1_. _________
Meihui ba Jieyi touched. _________
(41) [${}_{{\text{NP}}_{1}}$ 美惠] 把 [${}_{{\text{NP}}_{2}}$ 洁怡] 打动了_IC-1_. 她 _________ [IC-1, Pronoun]
[${}_{{\text{NP}}_{1}}$ Meihui] ba [${}_{{\text{NP}}_{2}}$ Jieyi] dadong-le_IC-1_. Ta _________
Meihui ba Jieyi touched. She _________
(42) [${}_{{\text{NP}}_{1}}$ 美惠] 把 [${}_{{\text{NP}}_{2}}$ 洁怡] 打动了_IC-1_. _________ [IC-2, Free]
[${}_{{\text{NP}}_{1}}$ Meihui] bei [${}_{{\text{NP}}_{2}}$ Jieyi] jiegu-le_IC-2_. _________
Meihui ba Jieyi fired. _________
(43) [${}_{{\text{NP}}_{1}}$ 美惠] 把 [${}_{{\text{NP}}_{2}}$ 洁怡] 打动了_IC-1_. 她 _________ [IC-2, Pronoun]
[${}_{{\text{NP}}_{1}}$ Meihui] ba [${}_{{\text{NP}}_{2}}$ Jieyi] jiegu-le_IC-2xs_. Ta _________
Meihui ba Jieyi fired. She _________

Participants wrote passage continuations using the same web interface used in Experiment 2.

#### Coding

The coding procedure used the same criteria as in Experiment 2.

### Results and discussion

We only included continuations about NP_1_ or NP_2_ for analysis. Continuations were excluded if the next-mentioned referent was coded as *unclear* (4.1%), *both* (5.4%) or *other* (2.8%). Continuations were also excluded if a reference to NP_1_ or NP_2_ was carried out using a referring expression other than a name, an overt pronoun, or a null pronoun (0.2%). This resulted in the exclusion of 12.5% of the total 3132 continuations, leaving a total of 2741 continuations in the dataset.

#### Next-mention biases

**Fig 11:**
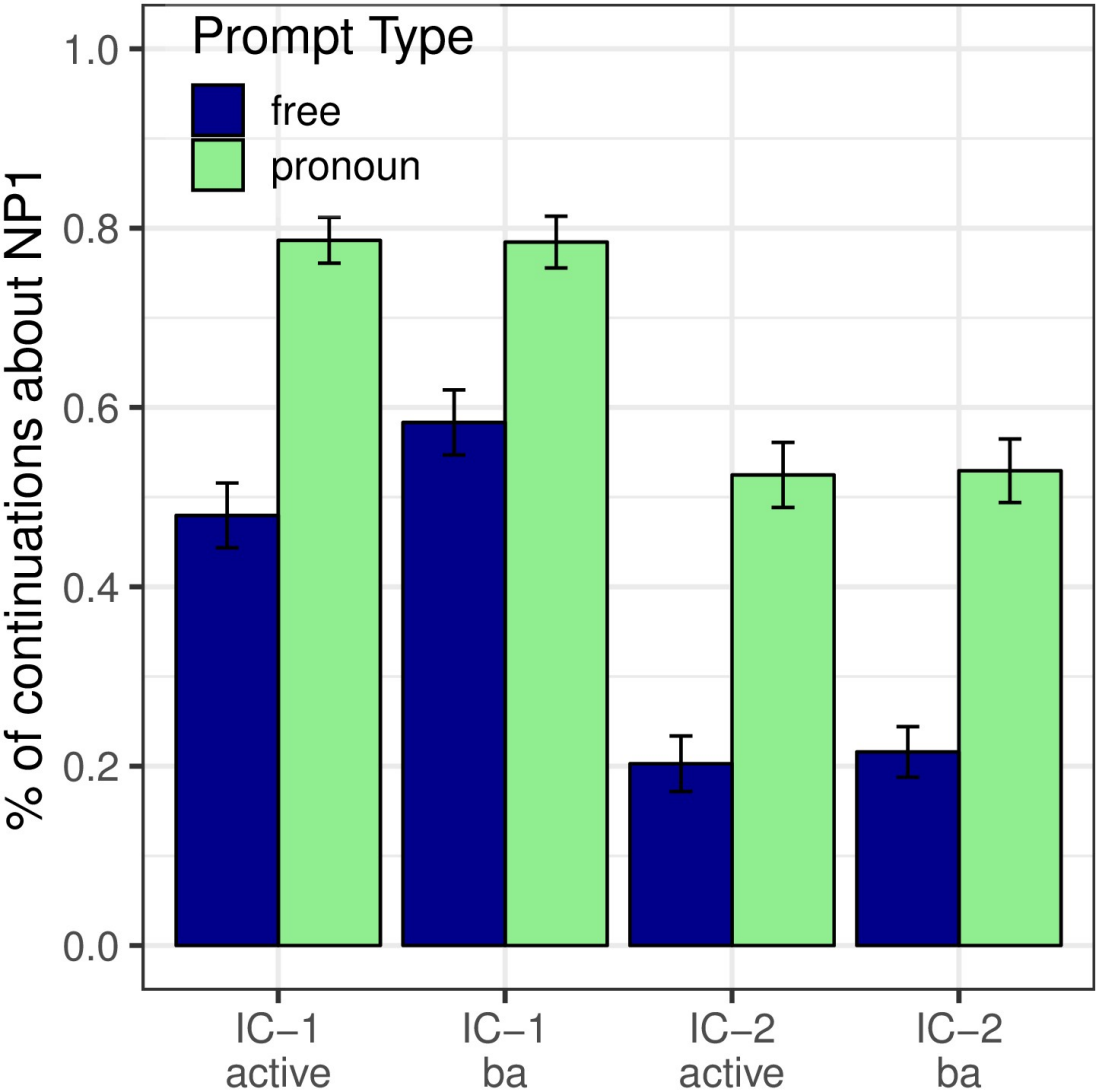
Expt 3: Proportion of continuations about the subject, by Verb Type, Prompt Type and syntactic construction.

Fig 11 shows the percentage of NP_1_ continuations by Syntactic Construction and Verb Type. Analyses show a main effect of Verb Type (*p* < 0.001): IC-1 verbs elicited more NP_1_ continuations than IC-2 verbs. There was also a main effect of Syntactic Construction (*p* = 0.017), whereby overall sentences with *ba* elicited more NP_1_ continuations than their non-*ba* counterparts. The interaction between Verb Type and Syntactic Construction was not significant (*p* = 0.339). Pairwise comparisons show that in IC-1 items, *ba* sentences elicited more NP_1_ continuations than canonical sentences (*p* = 0.033), whereas there was no significant difference in IC-2 items (*p* = 0.1).

**Fig 12:**
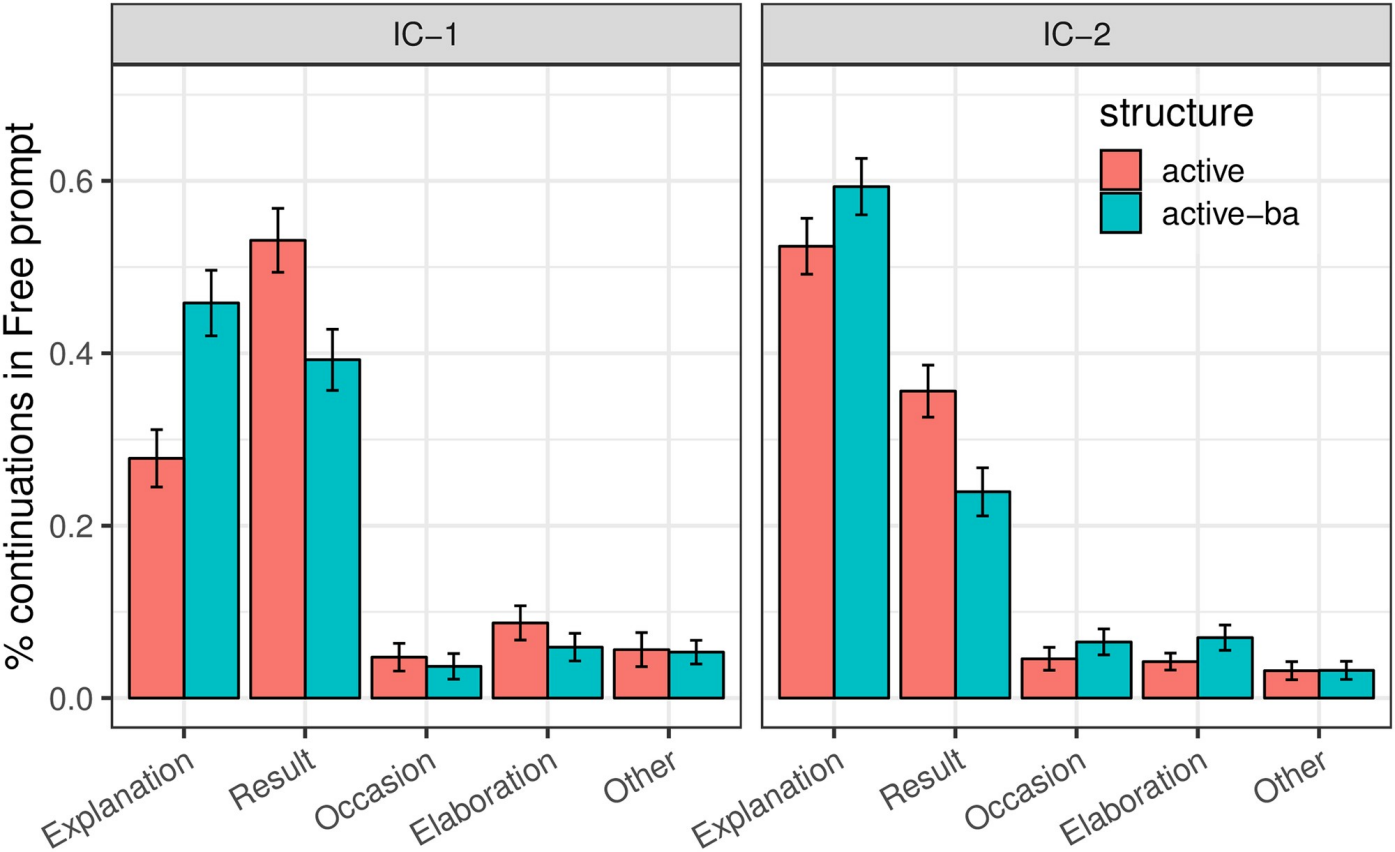
Expt 3: Distribution of coherence relations in free Prompt condition.

We now examine whether the *ba* construction affects the distribution of Coherence Relations with Verb Type as a controlled factor. Fig 12 shows the distribution of Coherence Relations in IC-1 and IC-2 items. Analyses show that there was a main effect of Syntactic Construction (*p* < 0.001), whereby *ba* constructions elicited more Explanation continuations than their canonical counterparts. There was also a main effect of Verb Type (*p* < 0.001), whereby overall IC-2 verbs had more Explanation continuations than IC-1 verbs. The interaction between Syntactic Construction and Verb Type was not significant (*p* = 0.267). Notably, syntactic construction affects the distribution of coherence relations for the IC-2 condition (Fig 12) despite the fact that it does not affect the distribution of next mention in the IC-2 condition (Fig 11). This pattern is theoretically possible since both Explanation and Result relations are object biased in IC-2 contexts. However, following up on our discussion of model dependencies in Experiment 2, the pattern is only consistent with a model in which syntactic construction directly affects coherence relations, as opposed to one in which their dependence is exclusively mediated by next mention.

**Fig 13:**
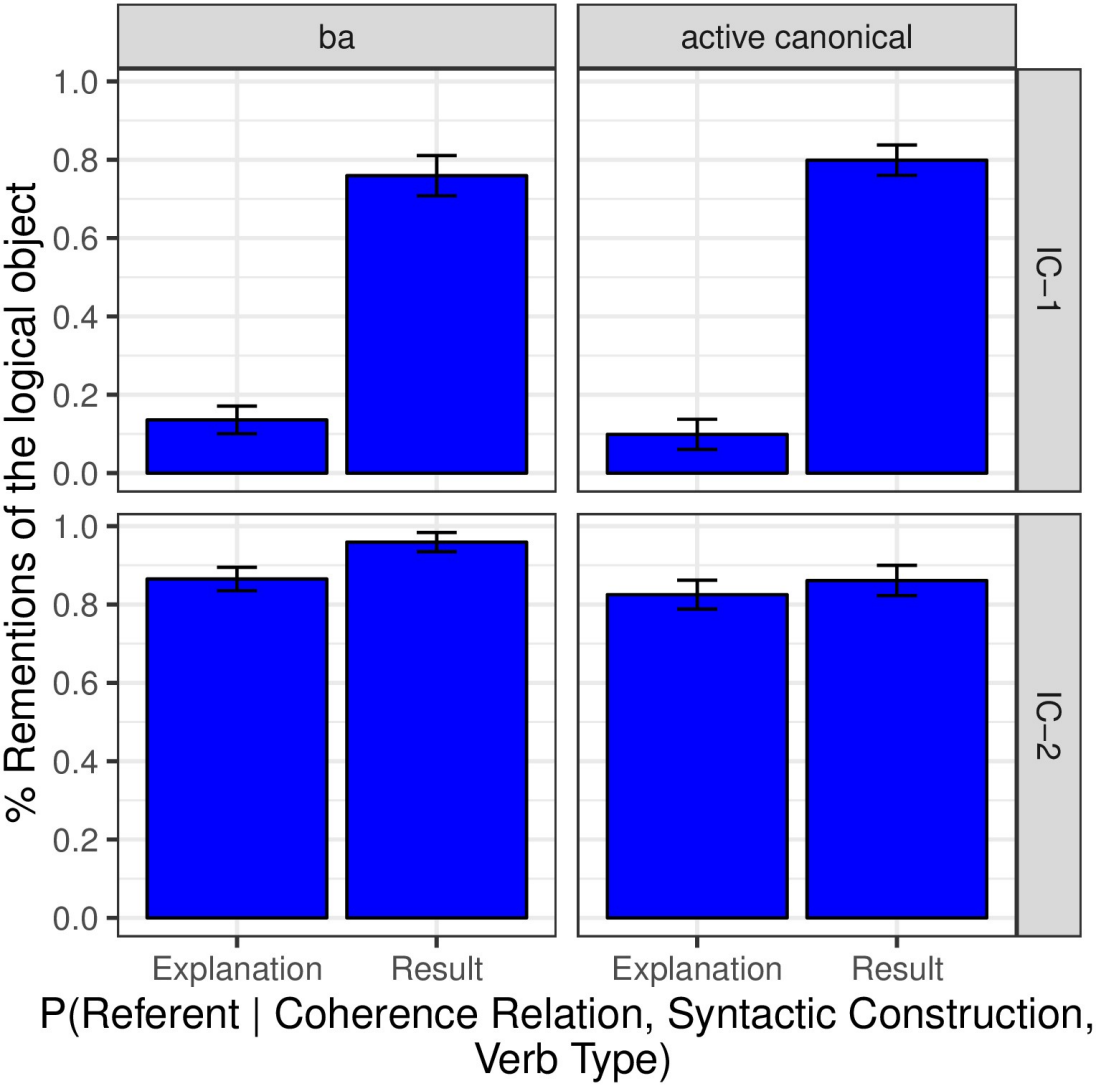
Expt 3: Next-mention biases in explanation and result coherence relations by Verb Type and by syntactic construction.

Next we examine whether the difference in Syntactic Construction affects next-mention biases when Coherence Relation is kept constant and Verb Type is controlled for. The answer is no. As seen in Fig 13, there was a main effect of Coherence Relation for both IC-1 (*p* < 0.001) and IC-2 (*p* < 0.001) items, whereby Explanation co-occurred with more NP_1_ continuations than Result. There was no effect of Syntactic Construction on next-mention biases.

#### Production biases

**Fig 14:**
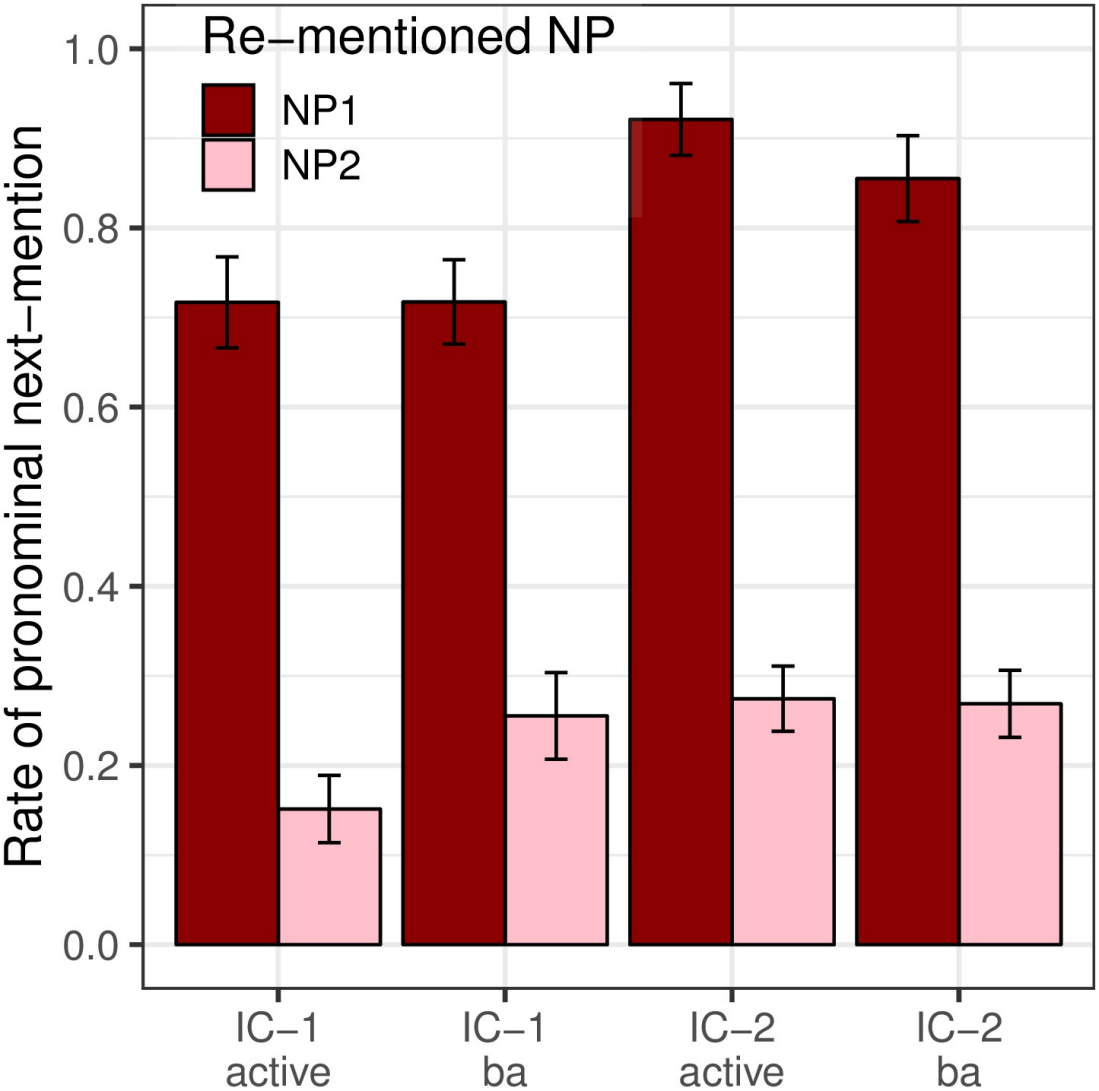
Expt 3: Rate of pronominalization, by Verb Type, Re-mentioned NP and syntactic construction.

Fig 14 illustrates the rate of pronominalization when Re-mentioned NP is conditioned on and null and overt pronouns are collapsed. Analyses show that there was main effect of Re-mentioned NP (*p*_*MCMC*_ < 0.001), with NP_1_ re-mentions significantly more likely to be pronominalized than NP_2_ re-mentions. As was found in Experiment 1, there was a main effect of Verb Type (*p*_*MCMC*_ = 0.002), with IC-2 verbs eliciting a greater rate of pronominalization than IC-1 verbs. There was no effect of Syntactic Construction (*p*_*MCMC*_ = 0.720), and no interaction between Re-mentioned NP and Syntactic Construction (*p*_*MCMC*_ = 0.224), Re-mentioned NP and Verb Type (*p*_*MCMC*_ = 0.098), or Syntactic Construction and Verb Type (*p*_*MCMC*_ = 0.116). There was no three-way interaction either (*p*_*MCMC*_ = 0.769).

The lack of interaction between Re-mentioned NP and Verb Type again confirms a key prediction of the strong Bayesian hypothesis. The prediction of the Topichood Hypothesis (contingent on the assumption that *ba* marks a secondary topic), however, is not confirmed: NP2 rementions are not more likely to be pronominalized in the *ba* condition than in the active condition (*p* = 0.847), based on pairwise comparison for NP2 pronominalization rate in the two different constructions (and the lack of interaction between Syntactic Construction and Re-mentioned NP).

#### Interpretation biases

Now we examine interpretation biases and compare them to next-mention biases. As shown in Fig 11, there was a main effect of Prompt Type (*p*_*MCMC*_ < 0.001), with the proportion of continuations about NP_1_ in the Pronoun Prompt condition higher than that in the Free Prompt condition. There was also a main effect of Verb Type (*p*_*MCMC*_ < 0.001), whereby IC-1 verbs elicited more NP_1_ continuations than IC-2 verbs. There was no main effect of Syntactic Construction (*p*_*MCMC*_ = 0.122), and no interaction between Prompt Type and Syntactic Construction (*p*_*MCMC*_ = 0.08), Prompt Type and Verb Type (*p*_*MCMC*_ = 0.122), or Syntactic Construction and Verb Type (*p*_*MCMC*_ = 0.671). The three-way interaction was not significant (*p*_*MCMC*_ = 0.340). These results are consistent with both the strong and the weak versions of the Bayesian hypothesis.

#### Model comparisons

**Fig 15:**
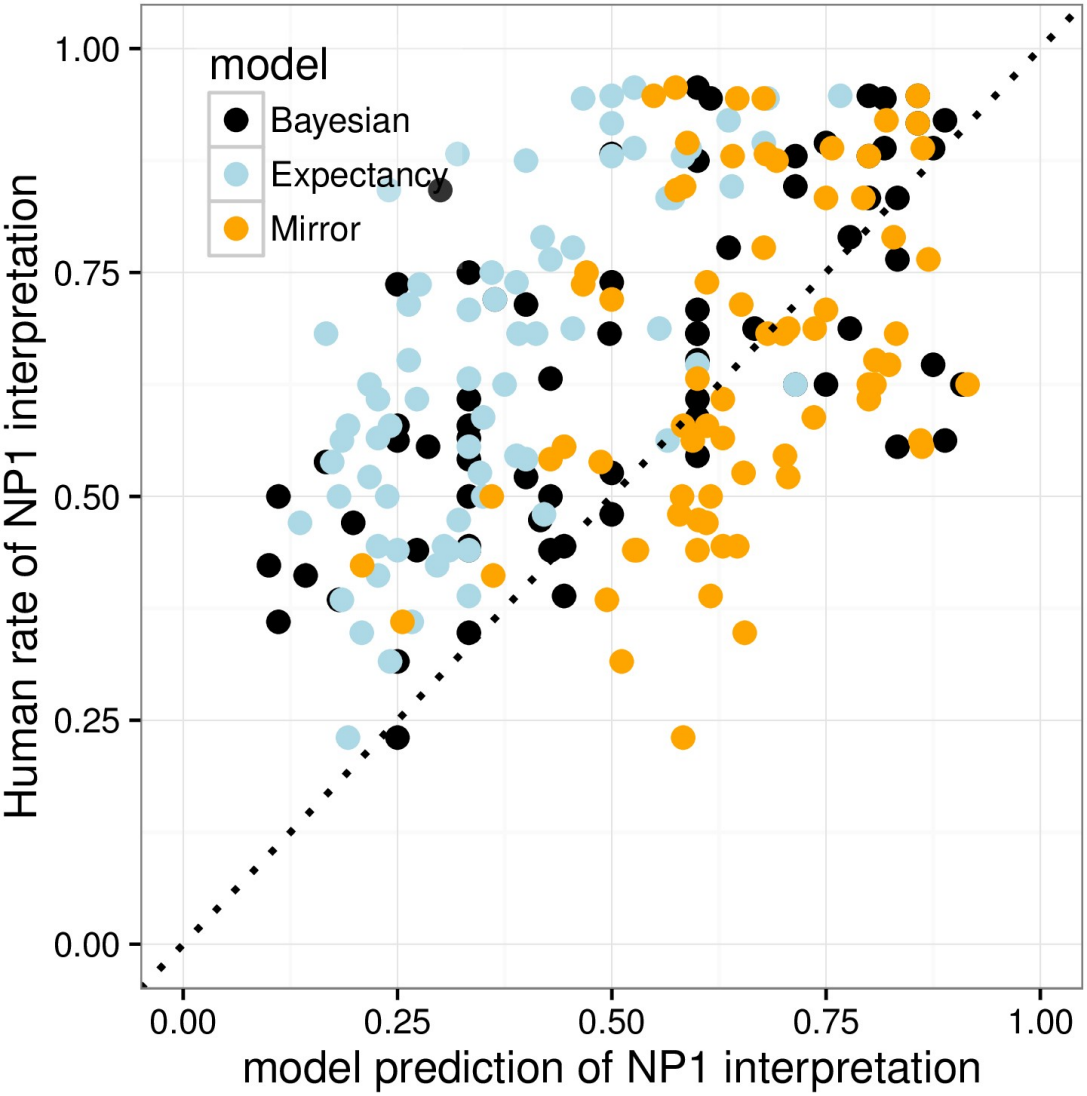
Expt 3: Item-by-item quantitative model evaluation for Experiment 3.

Fig 15 and Table 3 show the performance of the three models. Consistent with the previous experiments, the Mirror model substantially underpredicts cross-item variability in interpretation preferences, and the Expectancy model substantially underpredicts the likelihood-derived preference toward NP_1_ interpretations. The Bayesian model is the best of the three models on *R*^2^ and second-best on MSE and ACE; as in both previous experiments, the Expectancy model is the worst of the three models on MSE and ACE and the Mirror model is the worst of the three on *R*^2^. We performed paired t-tests on by-item squared error and cross-entropy (see Eqs (E1) and (E2)) between the Bayesian model and the other two models. For MSE, the Bayesian model performed significantly better than the Expectancy model (*p* < 0.001) but marginally worse than the Mirror model (*p* = 0.051). For ACE, the Bayesian model significantly outperformed the Expectancy model (*p* < 0.001), but significantly worse than the Mirror model (*p* = 0.043).

**Table 3:** Expt 3: R-squared, MSE, and ACE of the three models.

	Bayesian	Expectancy	Mirror
*R*^2^	**0.456**	0.451	0.186
*MSE*	0.046	0.092	**0.032**
*ACE*	0.990	1.128	**0.937**

MSE = Mean squared error; ACE = Average cross entropy.

We conjecture that the slightly worse performance of the Bayesian model on MSE and ACE in comparison with the Mirror model may be because its predictions involve two noisy estimates, compared to the Mirror model’s one. However, there are also some signs, also evident in the data of Experiments 1 and 2, that the Bayesian model may slightly underpredict the strength of participants’ NP_1_ interpretive preferences across the board, which would also hurt the Bayesian model on MSE and ACE.

### Summary

The results of Experiment 3 suggest that *ba*-active context sentences lead to a slightly higher rate of next-mentions of the subject than canonical active sentences in Mandarin Chinese. This effect is surprising since, if the *ba*-active were to have any effect on next-mention, one might expect that syntactically promoting the direct object would result in more, and not fewer, object mentions. However, this effect only reached significance in IC-1 contexts, and seems to have been completely mediated by coherence relations: *ba* elicited more Explanation continuations than the canonical active, and Explanation continuations are strongly biased toward NP_1_ for IC- 1 verbs. Notably, *ba* has no correlation with next-mention preferences once coherence relation is conditioned on. So, although it remains unclear why *ba* predisposes participants toward Explanation continuations, the effect on reference appears to be derivative.

As in Experiments 1 and 2, the results again showed that the grammatical role from which the referent is mentioned—subject vs. object—has a strong effect on the likelihood of pronominalization, and that this effect in turn influences pronoun interpretation biases. Regarding the Topichood Hypothesis, this experiment shows no confirmatory evidence. If *ba* marks a secondary topic and topics are more likely to be pronominalized than non-topics (controlling for grammatical function), then the Topichood Hypothesis predicts that NP2 rementions (but not NP1 rementions) should more likely be pronominalized in the *ba* condition than in the active condition. In our data, NP2 remention rates do not differ significantly across these two conditions. This implies either that *ba* does not mark a secondary topic [28] or that this prediction of the Topichood Hypothesis is incorrect, at least for secondary topics.

## General discussion

We conducted three experiments to evaluate whether pronoun production, interpretation, and the relationship between them in Mandarin Chinese is adequately captured by the Bayesian probabilistic framework [13, 14], according to which comprehenders reverse-engineer the speaker’s intended referent by way of Bayesian principles. Experiment 1 consisted of a baseline study that manipulated verb bias (subject vs. object biased IC verbs) and prompt type (free vs. pronoun). Experiment 2 explored the effects of syntactic structure by adding a condition that compared context sentences in the passive (the *bei* construction) with those in the canonical active. Experiment 3 explored the effect of promoting the grammatical object to a more topical position by comparing the *ba* active and canonical active constructions. As an ensemble, the results largely supported the predictions of the Bayesian model.

### Next-mention biases and coherence relations

The results concerning effects on next-mention biases came out generally as predicted. First, in all three experiments, the verb type manipulation had the expected effect on next-mention biases, whereby contexts that contain IC-1 verbs yielded more next mentions of the preceding subject than contexts that contain IC-2 verbs. This finding was not a surprise, especially in light of the fact that the verbs were selected based on the norming study described in the passage completion task section. The result nonetheless constitutes an important sanity check on the interpretation of the other findings, particularly with respect to predicted null effects of verb type on production.

In Experiment 2, we found that passivization increased the rate of re-mentions of the logical object. We hypothesize that this result, which is different than that found in English [10], arises in part from the affectedness imputed by the Mandarin passive to the logical object: participants produced more Result continuations after passives than after canonical actives, and Result continuations are predominately about the logical object.

Experiment 3 yielded an unanticipated finding whereby the *ba* construction led to more continuations about the preceding subject NP_1_ than canonical actives, but only in IC- 1 contexts. This finding is also explicable through the mediating role of coherence relations: *ba* led to more Explanation continuations than canonical actives, and for IC- 1 verbs (but not IC- 2 verbs) Explanations usually involve NP_1_ re-mentions. Although we did not anticipate this result, recall that a previous study [37] found a similar effect for IC-1 verbs. A preliminary analysis of our data suggests that the effect might be due to there being a greater number of Explanation continuations in the *ba* active condition, but the reasons why this would be the case remain unclear and require further investigation.

### Production biases, grammatical role, and topichood

The core predictions concerning production biases were also confirmed. Recall that the strong form of the Bayesian model predicts that grammatical role will have an effect on production biases, but that verb type will not. The predicted effect of grammatical role was found in all three experiments, whereby subject mentions were pronominalized significantly more often than object mentions. Consistent with the strong form of the Bayesian hypothesis, there was no interaction between grammatical role and verb type.

A prediction of the Topichood Hypothesis regarding the primacy of topichood over grammatical role, however, was not confirmed. Recall that previous work [10] found that mentions of the subject of passive clauses were pronominalized more often than subjects of active clauses, suggesting that pronominalization biases are dependent primarily on topichood, and hence only indirectly on grammatical role. The results of our Experiment 2, however, found that mentions of the subject in the passive condition were not pronominalized at a greater rate than those in the active condition. Further, Experiment 3 found that promoting the direct object to a preverbal position using the *ba* construction did not result in a greater rate of pronominalization as compared to postverbal objects in the canonical active construction, suggesting that either *ba* does not mark secondary topics [28] or that this prediction of the Topichood Hypothesis is incorrect, at least for secondary topics.

### Interpretation biases

Finally, the core predictions of both the weak and the strong forms of the Bayesian model regarding the relationship between pronoun interpretation and production were also confirmed. According to both, when comprehenders encounter a pronoun they attempt to reverse-engineer the speaker’s intended referent through Bayesian principles. One therefore expects to see influences of both next mention biases (as represented by the prior) and production biases (as represented by the likelihood) on interpretation biases. These predictions were confirmed in all three experiments, where the differences in next mention biases created by the verb type manipulation revealed themselves by way of an effect on interpretation biases. Furthermore, interpretation biases were consistently skewed toward the previous subject as compared to the corresponding next-mention biases, revealing the predicted effect of production biases as well.

We also compared quantitative item-by-item predictions of the Bayesian model and two other models, the Expectancy and Mirror models, that could be taken to formally instantiate competing theories in the literature. We used Free Prompt condition data to estimate the predictions of the three models, and used three metrics—*R*^2^, MSE, and ACE—to evaluate model performance. In general, the Bayesian model performed better according to the three metrics than two competing models derived from the literature: in nine total evaluations, the Bayesian model scored best in six, and second best in three. In general, the Expectancy model underpredicted the strength of the bias to interpret a pronoun as coreferring with the preceding subject NP_1_. This pattern is evident in Figs 3, 10 and 15 as a tendency for the Expectancy model points to sit above the *x* = *y* perfect-prediction line—a pattern that is itself predicted by the Bayesian model due to the Expectancy model’s omission of the likelihood term, which biases pronouns toward preceding-subject NP_1_ interpretations. Conversely, the Mirror model tended to underpredict cross-item variability in interpretation preferences. This effect is evident in the tendency for Mirror model points to cluster tightly just to the right of the *x* = 0.5 center, regardless of the actual item-specific interpretive bias—a pattern predicted by the Bayesian model because the Mirror model omits the influence of the prior, which is more variable across items than the likelihood.

These limitations of the Expectancy and Mirror models notwithstanding, the fit between Bayesian model predictions and observed interpretive preferences is not perfect either. To some extent the imperfect fit may simply reflect estimation error in determining model predictions, which affects the Bayesian model in both prior $\hat{P}\left(\text{referent}\right)$ and likelihood $\hat{P}\left(\text{pronoun}|\text{referent}\right)$ estimation. But in addition to noise in model predictions, the Bayesian model also tended to underpredict the interpretive bias toward the previous subject NP_1_, albeit not to the extent of the Expectancy model. This limitation might suggest a systematic deviation from normative probabilistic inference on the part of human comprehenders that we would also find in naturalistic language usage, or it could be the result of some other, unforeseen cause that is more specific to this particular task. Future work will be required to examine the reasons why our experimental participants may not always have perfectly executed normative probabilistic reasoning. Nevertheless, the factorial and model-comparison analyses of our experimental data provide clear evidence of all the key qualitative signatures of Bayesian inference in Mandarin pronoun interpretation.

## Conclusion

To sum, three passage completion experiments in Mandarin Chinese provided broad support for the Bayesian model of pronoun interpretation. As predicted, the data revealed a dissociation between the factors that determine pronoun production and pronoun interpretation: interpretation biases do not straightforwardly mirror expectations about next-mention, nor do they simply mirror pronoun production biases. Rather, interpretation biases reflect the joint influence of next-mention biases and production biases: the comprehender reasons about the message that the speaker intends to convey as well as her linguistic choices in conveying that message. Comparisons between the full Bayesian model and two variants show that overall the full Bayesian model best predicts human behavior in pronoun interpretation. These findings are broadly consistent with previous studies in English [10, 14], but are inconsistent with theories that posit (or otherwise implicitly assume) that a unitary notion of entity salience mediates between production and interpretation.

Our results mostly support the tenets of the strong form of Bayesian hypothesis in addition to the weak form: next-mention biases are primarily affected by semantic and pragmatic factors (verb type, coherence relations) whereas production biases are primarily affected by the grammatical role of the intended referent. At the same time, however, they call into question the hypothesis that topichood, rather than grammatical role, determines production biases [10]. Unlike their study, two syntactic manipulations in Mandarin that are hypothesized to affect the topichood of referents—promotion of the surface subject via the *bei* passive, and promotion of the object via the *ba* active—did not affect rate of pronoun production. Further work will be required to understand the reasons for the different results across these studies.

We also found that passivization increased the next-mention rate for the logical object. We hypothesize that this increase originates in part from the affectedness introduced by the *bei* construction, which in turn led participants to write a greater number of Result continuations about the logical object. Whereas these findings are specific to Mandarin Chinese, the increased next-mention biases for the logical object were reflected in the corresponding pronoun interpretation biases, as predicted by the Bayesian model.

The successful application of the Bayesian model holds important lessons for future cross-linguistic work on pronoun interpretation. Whereas pronoun interpretation in a wide variety of languages has been investigated and reported on in the literature, it has generally been examined though the lens of the traditional view described in the introduction (or without considering the relationship between interpretation and production at all), according to which there is a single notion of entity salience that mediates between pronoun production and interpretation. This view has yielded a standard approach to analyzing reference behavior that, if the Bayesian model proves to be correct, will need to be revised in at least three ways.

First, the Bayesian model makes it clear that understanding pronoun interpretation and production requires identifying not only what contextual factors affect them, but also the structure that governs their influence [48]. Research pursued under the traditional view has tended to analyze various factors—grammatical role, grammatical parallelism, thematic role, information structure, semantics, world knowledge, and so forth—as an unstructured “bag of cues”. This approach has historically seemed reasonable since, on the traditional view, the discourse theorist’s job is primarily to identify the factors that affect the unitary notion of salience that the view takes for granted.

On the Bayesian model, however, a structured set of dependencies relate the different contextual factors, such that all factors ultimately influence pronoun interpretation only indirectly, by way of their influence on either the prior (next-mention biases) or the likelihood (production biases). This means that not all factors that *affect* pronoun interpretation necessarily belong in one’s *theory* of pronouns. To take an example, the results of the pronoun prompt conditions in our experiments could be interpreted as demonstrating that verb type (IC-1 vs. IC-2) has a direct effect on pronoun biases, as previous work has claimed based on similar studies in English and other languages. However, examination of the data collected in the free prompt condition reveals that the influence of verb type is only indirect: verb type influences the prior probability of next mention, which in turn influences pronoun interpretation. The production bias—i.e. the degree of preference for pronominalizing a given referent—is by and large not affected. This suggests that verb type, along with any of a number of other factors that only affect the probability of next mention, should not be included in one’s theory of pronoun interpretation; these factors exert their influence on the discourse context independently of whether the addressee encounters a pronoun at any particular point in the discourse. As far as pronoun interpretation is concerned, the probability distribution associated with next mention is a black box.

Second, the Bayesian model clarifies the appropriate baseline to measure the interpretation biases contributed by the occurrence of a pronoun. Examinations of pronoun interpretation under the traditional view have typically compared measured biases against the uniform distribution, i.e. a 50/50 split when there are two competing referents made available by the context. On this view, for instance, one might look at the results of our first experiment and conclude that pronouns are “subject biased” in IC-1 contexts and “object biased” in IC-2 contexts, since the measured biases are typically above and below the 50% break point respectively. Whereas this interpretation may seem sensible on the traditional view, it is not on the Bayesian model. According to the Bayesian model, the contribution of a pronoun is the difference between the interpreter’s expectations about who will be mentioned next *before* the pronoun is encountered (the prior, as measured by the free prompt conditions in our experiments) and the probability distribution regarding mention *after* a pronoun is encountered (the posterior, as measured by the pronoun prompt conditions). It is therefore the prior that provides the appropriate baseline which with to measure the contribution made by a pronoun. In the experiments reported on here, as well as all others run in the last author’s laboratory, pronouns *always* contribute a bias toward the subject as compared to the free prompt condition, even if the ultimate interpretation bias points away from the subject. That being said, the full range and typological distribution of interpretation biases offered by the pronoun inventories of the world’s languages remains an open question. The Bayesian model makes testable predictions about the relationship between next-mention expectations, pronoun production, and pronoun interpretation, and insofar as these predictions continue to be confirmed in future cross-linguistic work, the model clarifies the terms on which the range and distribution of interpretation biases can be assessed.

Finally, and relatedly, the estimation of the prior in the Bayesian model offers a natural control for the item-specific biases associated with experimental stimuli. As argued in Kehler and Rohde [14], there has been a persistent methodological problem in the literature with respect to stimulus creation, whereby interpretation biases are evaluated using a set of stimuli that have been constructed to seem intuitively unobjectionable (‘pragmatically unbiased’) but with no independent verification. Because pronoun interpretation biases on the Bayesian model are measured against the prior, the inclusion of free prompt conditions in experiments yields fine-grained quantitative measurements of the degree of bias associated with particular contexts. Further, on this model there is no need to consider IC contexts as being of a qualitatively different sort than others: when measuring the prior across context types, it quickly becomes clear that *all* contexts are associated with some amount of bias. IC contexts are perhaps notable only because their biases are stronger than those found in many other contexts.

We therefore argue that a proper cross-linguistic analysis of pronominal reference will require that a broad range of languages be revisited experimentally, to include passage completion studies conducted with both pronoun prompt and free prompt conditions, since only then can the predictions of the Bayesian and competing models be properly evaluated. We hope that the studies reported on here will inspire further work in this direction.

## Supporting information

S1 Appendix Experimental stimuli for the three experiments. Each experiment has 36 items.
(PDF)
