# Supplementary material for: Pronoun interpretation in Mandarin Chinese follows principles of Bayesian inference
Source: PLoS One. 2020 Aug 19;15(8):e0237012. doi: 10.1371/journal.pone.0237012 (PMC7446932; doi:10.1371/journal.pone.0237012)
Supplement: S1 Appendix — Each experiment has 36 items. (PDF) [file pone.0237012.s001.pdf]

Appendix

Experimental Stimuli for Experiment 1

IC-1 items

1. 诗音迷惑了静宜。（她）…… *"Shiyin confused Jingyi. (She) ..."*
2. 孟轩感动了俊新。（他）…… *"Mengxuan moved Junxin. (He) ..."*
3. 淑贞激励了香惠。（她）…… *"Shuzhen encouraged Xianghui. (She) ..."*
4. 永芸惊动了韵梅。（她）…… *"Yongyun startled Yunmei. (She) ..."*
5. 志浩打动了修铭。（他）…… *"Zhihao touched Xiuming. (He) ..."*
6. 克军出卖了宏林。（他）…… *"Kejun betrayed Honglin. (He) ..."*
7. 佩锦吓坏了思琪。（她）…… *"Peijin scared Siqi. (She) ..."*
8. 志成激怒了山明。（他）…… *"Zhicheng angered Shanming. (He) ..."*
9. 丽丽欺骗了依琳。（她）…… *"Lili cheated Yilin. (She) .."*
10. 唐军惹恼了赵力。（他）…… *"Tanjun annoyed Zhaoli. (He) ..."*
11. 佳琳逗笑了金凤。（她）…… *"Jialin amused Jinfeng. (She) ..."*
12. 建延迷住了国旭。（他）…… *"Jianyan charmed Guoxun. (He) ..."*
13. 许玲冒犯了蔡琪。（她）…… *"Xuling offended Caiqi. (She) ..."*
14. 容峰侮辱了智钧。（他）…… *"Rongfeng humiliated Zhijun. (He) ..."*
15. 佳玲吸引了姿莹。（她）…… *"Jianling attracted Ziying. (She) ..."*
16. 吴龙辜负了陈杰。（他）…… *"Wulong disappointed Chenjie. (He) ..."*

IC-2 items

1. 美惠批评了洁怡一顿。（她）...... *"Meihui criticized Jieyi. (She)..."*
2. 建志责备了政宇一通。（他）...... *"Jianzhi faulted Zhiyu. (He)..."*
3. 淑玲嘲笑了雅琪一番。（她）...... *"Shuling mocked Yaqi. (She)..."*
4. 俊杰表扬了家豪一番。（他）...... *"Junjie praised Jiahao. (He)..."*
5. 秋萍责骂了玉婷一通。（她）...... *"Qiuping scolded Yuting. (She) ..."*
6. 振华称赞了于勇一番。（他）...... *"Zhenhua acclaimed Yuyong. (He) ..."*
7. 宛珠夸奖了雅慧一番。（她）...... *"Wanzhu complimented Yahui. (She)..."*
8. 建铭教训了勇志一顿。（他）...... *"Jianming disciplined Yongzhi. (He)..."*
9. 芳妮谴责了秀玲一番。（她）...... *"Fangni condemned Xiuling. (She)..."*
10. 忠宪原谅了蒋平。（他）...... *"Zhongxian forgave Jiangping. (He) ..."*
11. 湘琳鄙视了紫芸。（她）...... *"Xiangling held Ziyun in contempt. (She) ..."*
12. 俊峰解雇了宗龙。（他）...... *"Junfeng fired Zonglong. (He) ..."*
13. 毅刚低估了子豪。（他）...... *"Yigang underestimated Zihao. (He) ..."*
14. 茵茵抓获了桂芝。（她）...... *"Yinyin captured Guizhi. (She) ..."*
15. 俊贤起诉了舜明。（他）...... *"Junxian sued Shunming. (He) ..."*
16. 佩芬投诉了郁花。（她）...... *"Pengfen made a complaint about Yuhua. (She) ..."*
17. 伟杰殴打了延东。（他）...... *"Weijie beat Yandong. (He) ..."*
18. 孟璇安慰了李月。（她）...... *"Mengxuan consoled Liyue. (She) ..."*

19. 柏豪埋怨了王康一顿。（他）...... *"Bohao blamed Wangkang. (He) ..."*

20. 阿美数落了杨娟一顿。（她）...... *"Amei reproached Yangjuan. (She) ..."*

Experimental Stimuli for Experiment 2

The items in the baseline condition is the same as in Experiment 1. The items in Experimental condition is listed as follows.

IC-1 items

1. 静宜被诗音迷惑了。（她）...... *"Jingyi was confused by Shiyin. (She) ..."*
2. 俊新被孟轩感动了。（他）...... *"Junxin was moved by Mengxuan. (He) ..."*
3. 香惠被淑贞激励了。（她）...... *"Xianghui was encouraged by Shuzhen. (She) ..."*
4. 韵梅被永芸惊动了。（她）...... *"Yunmei was startled by Yongyun. (She) ..."*
5. 修铭被志浩打动了。（他）...... *"Xiuming was touched by Zhihao. (He) ..."*
6. 宏林被克军出卖了。（他）...... *"Hongling was betrayed by Kejun. (He) ..."*
7. 思琪被佩锦吓坏了。（她）...... *"Siqi was scared by Peijin. (She) ..."*
8. 山明被志成激怒了。（他）...... *"Shanming was angered by Zhicheng. (He) ..."*
9. 依琳被丽丽欺骗了。（她）...... *"Yilin was cheated by Lili. (She) .."*
10. 赵力被唐军惹恼了。（他）...... *"Zhaoli was annoyed by Tangjun. (He) ..."*
11. 金凤被佳琳逗笑了。（她）...... *"Jinfeng was amused by Jialin. (She) ..."*

12. 国旭被建延迷住了。（他）...... *"Guoxuan was charmed by Jianyan. (He) ..."*

13. 蔡琪被许玲冒犯了。（她）...... *"Caiqi was offended by Xuling. (She) ..."*

14. 智钧被容峰侮辱了。（他）...... *"Zhijun was humiliated by Rongfeng. (He) ..."*

15. 姿莹被佳玲吸引了。（她）...... *"Ziying was attracted by Jialing. (She) ..."*

16. 陈杰被吴龙辜负了。（他）...... *"Chenjie was disappointed by Wulong. (He) ..."*

IC-2 items

1. 洁怡被美惠批评了一顿。（她）...... *"Jieyi was criticized by Meihui. (She)..."*

2. 政宇被建志责备了一通。（他）...... *"Zhiyu was faulted by Jianzhi. (He)..."*

3. 雅琪被淑玲嘲笑了一番。（她）...... *"Yaqi was mocked by Shuling. (She)..."*

4. 家豪被俊杰表扬了一番。（他）...... *"Jiahao was praised by Junjie. (He)..."*

5. 玉婷被秋萍责骂了一通。（她）...... *"Yuting was scolded by Qiuping. (She) ..."*

6. 于勇被振华称赞了一番。（他）...... *"Yuyong was acclaimed Zhenhua. (He) ..."*

7. 雅慧被宛珠夸奖了一番。（她）...... *"Yahui was complimented by Wanzhu. (She)..."*

8. 勇志被建铭教训了一顿。（他）...... *"Yongzhi was disciplined by Jianming. (He)..."*

9. 秀玲被芳妮谴责了一番。（她）...... *"Xiuling was condemned by Fangni. (She)..."*

10. 蒋平被忠宪原谅了。（他）...... *"Jiangping was forgiven by Zhongxian. (He) ..."*

11. 紫芸被湘琳鄙视了。（她）...... *"Ziyun was held in contempt by Xiangling. (She) ..."*

12. 宗龙被俊峰解雇了。（他）...... *"Zonglong was fired by Junfeng. (He) ..."*

13. 子豪被毅刚低估了。（他）...... *"Zihao was underestimated by Yigang. (He) ..."*

14. 桂芝被茵茵抓获了。（她）...... *"Guizhi was captured by Yinyin. (She) ..."*

15. 舜明被俊贤起诉了。（他）...... *"Shuming was sued by Junxian. (He) ..."*（他）......

16. 郁花被佩芬投诉了。（她）...... *"Yuhua was complained by Peifen. (She) ..."*

17. 延东被伟杰殴打了。（他）...... *"Yandong was beaten by Weijie. (He) ..."*

18. 李月被孟璇小看了。（她）...... *"Liyue was looked down upon by Mengxuan. (She) ..."*

19. 王康被柏豪埋怨了一顿。（他）...... *"Wangkang was blamed by Bohao. (He) ..."*

20. 杨娟被阿美数落了一顿。（她）...... *"Yangjuan was reproached by Amei. (She) ..."*

Experimental Stimuli for Experiment 3

The items in the baseline condition is the same as in Experiment 1. The items in Experimental condition is listed as follows.

IC-1 items

1. 诗音把静宜迷惑了。（她）...... *"Shiyin confused Jingyi. (She) ..."*

2. 孟轩把俊新感动了。（他）...... *"Mengxuan moved Junxin. (He) ..."*

3. 淑贞把香惠激励了。（她）...... *"Shuzhen encouraged Xianghui. (She) ..."*
4. 永芸把韵梅恐吓了。（她）...... *"Yongyun startled Yunmei. (She) ..."*
5. 志浩把修铭打动了。（他）...... *"Zhihao touched Xiuming. (He) ..."*
6. 克军把宏林出卖了。（他）...... *"Kejun betrayed Honglin. (He) ..."*
7. 佩锦把思琪吓坏了。（她）...... *"Peijin scared Siqi. (She) ..."*
8. 志成把山明激怒了。（他）...... *"Zhicheng angered Shanming. (He) ..."*
9. 丽丽把依琳欺骗了。（她）...... *"Lili cheated Yilin. (She) .."*
10. 唐军把赵力惹恼了。（他）...... *"Tanjun annoyed Zhaoli. (He) ..."*
11. 佳琳把金凤逗笑了。（她）...... *"Jialin amused Jinfeng. (She) ..."*
12. 建延把国旭迷住了。（他）...... *"Jianyan charmed Guoxun. (He) ..."*
13. 许玲把蔡琪冒犯了。（她）...... *"Xuling offended Caiqi. (She) ..."*
14. 容峰把智钧侮辱了。（他）...... *"Rongfeng humiliated Zhijun. (He) ..."*
15. 佳玲把姿莹吸引了。（她）...... *"Jianling attracted Ziying. (She) ..."*
16. 吴龙把陈杰辜负了。（他）...... *"Wulong disappointed Chenjie. (He) ..."*

IC-2 items

1. 美惠把洁怡批评了一顿。（她）...... *"Meihui criticized Jieyi. (She)..."*
2. 建志把政宇责备了一通。（他）...... *"Jianzhi faulted Zhiyu. (He)..."*
3. 淑玲把雅琪嘲笑了一番。（她）...... *"Shuling mocked Yaqi. (She)..."*
4. 俊杰把家豪表扬了一番。（他）...... *"Junjie praised Jiahao. (He)..."*

5. 秋萍把玉婷责骂了一通。（她）…… *"Qiuping scolded Yuting. (She) ..."*

6. 振华把于勇称赞了一番。（他）…… *"Zhenhua acclaimed Yuyong. (He) ..."*

7. 宛珠把雅慧夸奖了一番。（她）…… *"Wanzhu complimented Yahui. (She)..."*

8. 建铭把勇志教训了一顿。（他）…… *"Jianming disciplined Yongzhi. (He)..."*

9. 芳妮把秀玲谴责了一番。（她）…… *"Fangni condemned Xiuling. (She)..."*

10. 忠宪把蒋平原谅了。（他）…… *"Zhongxian forgave Jiangping. (He) ..."*

11. 湘琳把紫芸鄙视了。（她）…… *"Xiangling held Ziyun in contempt. (She) ..."*

12. 俊峰把宗龙解雇了。（他）…… *"Junfeng fired Zonglong. (He) ..."*

13. 毅刚把子豪低估了。（他）…… *"Yigang underestimated Zihao. (He) ..."*

14. 茵茵把桂芝抓获了。（她）…… *"Yinyin captured Guizhi. (She) ..."*

15. 俊贤把舜明起诉了。（他）…… *"Junxian sued Shunming. (He) ..."*

16. 佩芬把郁花投诉了。（她）…… *"Pengfen made a complaint about Yuhua. (She) ..."*

17. 伟杰把延东殴打了。（他）…… *"Weijie beat Yandong. (He) ..."*

18. 孟璇把李月小看了。（她）…… *"Mengxuan looked down upon Liyue. (She) ..."*

19. 柏豪把王康埋怨了一顿。（他）…… *"Bohao blamed Wangkang. (He) ..."*

20. 阿美把杨娟数落了一顿。（她）…… *"Amei reproached Yangjuan. (She) ..."*
